# Ebola virus disrupts the inner blood-retinal barrier by induction of vascular endothelial growth factor in pericytes

**DOI:** 10.1371/journal.ppat.1011077

**Published:** 2023-01-18

**Authors:** Jiawang Gao, Zhengyuan Guo, Wei Li, Xiaowei Zhang, Xian-En Zhang, Zongqiang Cui

**Affiliations:** 1 State Key Laboratory of Virology, Wuhan Institute of Virology, Center for Biosafety Mega-Science, Chinese Academy of Sciences, Wuhan, China; 2 University of Chinese Academy of Sciences, Beijing, China; 3 Faculty of Synthetic Biology, Shenzhen Institutes of Advanced Technology, Chinese Academy of Sciences, Shenzhen, China; University of Pittsburgh, UNITED STATES

## Abstract

Ebola virus (EBOV) causes severe hemorrhagic fever in humans with high mortality. In Ebola virus disease (EVD) survivors, EBOV persistence in the eyes may break through the inner blood–retinal barrier (iBRB), leading to ocular complications and EVD recurrence. However, the mechanism by which EBOV affects the iBRB remains unclear. Here, we used the in vitro iBRB model to simulate EBOV in retinal tissue and found that Ebola virus-like particles (EBO-VLPs) could disrupt the iBRB. Cytokine screening revealed that EBO-VLPs stimulate pericytes to secrete vascular endothelial growth factor (VEGF) to cause iBRB breakdown. VEGF downregulates claudin-1 to disrupt the iBRB. Ebola glycoprotein is crucial for VEGF stimulation and iBRB breakdown. Furthermore, EBO-VLPs caused iBRB breakdown by stimulating VEGF in rats. This study provides a mechanistic insight into that EBOV disrupts the iBRB, which will assist in developing new strategies to treat EBOV persistence in EVD survivors.

## Author summary

Ebola virus (EBOV) persistence in eyes has been frequently reported and has become an enormous international public health challenge. EBOV persistence in the eyes may break through the inner blood–retinal–barrier (iBRB), leading to ocular complications and EBOV recurrence. However, the mechanism by which EBOV affects the iBRB remains unclear. Here, we found that Ebola virus-like particles stimulate retinal pericytes to secrete vascular endothelial growth factor (VEGF) to cause iBRB breakdown. VEGF causes iBRB breakdown by disrupting the tight junction protein claudin-1. Ebola glycoprotein plays a key role in VEGF stimulation and iBRB breakdown. Our study provides a mechanistic insight into that EBOV disrupts the iBRB, which will assist in developing new strategies to treat EBOV persistence in EBOV survivors.

## Introduction

Ebola virus (EBOV) is an enveloped, filamentous, nonsegmented negative-sense RNA virus in the family *Filoviridae* that causes a severe human viral hemorrhagic disease called Ebola virus disease (EVD), which has high morbidity and mortality [1]. Since the first outbreak in 1976, EBOV has been repeatedly reemerging to cause epidemics and has resulted in tens of thousands of EVD cases and deaths [2]. In EVD survivors, EBOV persistence has been frequently reported and has become an increasing concern [2–3].

EBOV persistence has been shown in immune-privileged sites, including the eyes, brain and testes [4–6]. Viral materials were detected in these immune-privileged sites in EVD survivors long after clinical recovery with no detectable viral material in blood or target organs [7]. EBOV persistence in immune-privileged sites also leads to EVD recurrence in some survivors [5]. This finding demonstrated that during persistence, EBOV can cross the blood-tissue barrier to enter the bloodstream from immune-privileged sites. EVD recurrence caused by the dissemination of EBOV from immune-privileged sites into the blood increases the risk of the initiation of new chains of viral transmission and causes an enormous international public health challenge [8–9].

EBOV persistence has been reported to cause various sequelae, such as arthralgia, cognitive impairment, headache, hearing loss, and myalgia, in a large number of EVD survivors [10]. Among them, approximately 35% of EVD survivors suffered from different kinds of ocular complications, including blurry vision, vitreous inflammation and optic neuropathy [11–12]. Uveitis and retinitis are the most common ocular symptoms in EVD survivors. Uveitis has been reported in 13–34% of EVD survivors [13]. Moderate to severe retinitis has been found in EBOV-infected rhesus monkey survivors, with the infiltration and accumulation of inflammatory cells in the retinal perivascular space and adjacent structures in the retina [14]. EBOV had been isolated from the eyes in EVD survivors [5]. Persistence in the eyes increased the risk of viral transmission during the ophthalmic treatment [15]. Due to the presence of the inner blood–retinal barrier (iBRB), inflammatory cells in blood cannot reach the retinal tissue and cause inflammation in healthy conditions [16]. The appearance of inflammatory cells in the perivascular space and adjacent structures in the retina of EVD survivors suggested iBRB breakdown during EBOV persistence.

The iBRB is crucial for the stability of the retinal microenvironment, which strictly restricts ions, proteins and other molecules in blood [17]. The iBRB is formed by the tight junctions between adjacent retinal capillary endothelial cells. Pericytes and astrocytes surround retinal endothelial cells to maintain barrier function through the release of cytokines and trophic factors in the retinal microenvironment [18–19]. Some viruses, such as human cytomegalovirus (HCMV), Zika virus and human immunodeficiency virus (HIV), have been reported to cause ocular diseases through the breakdown of the iBRB [20–22]. These viruses can cause iBRB breakdown by infecting endothelial cells and causing direct cytopathic effects or inducing cells in the iBRB to secrete cytokines, leading to endothelial dysfunction and an increase in iBRB permeability [23–24]. For EBOV, EVD recurrence and retinitis in EVD survivors strongly suggest that EBOV can cause iBRB breakdown in EBOV persistence. However, during persistence, how EBOV breaks through the iBRB to enter the bloodstream is still unclear.

Here, we examined the mechanism by which EBOV regulates the iBRB during persistence. By using in vitro barrier models to simulate EBOV in retinal tissue, we found that Ebola virus-like particles (EBO-VLPs), which consist of the VP40 protein and glycoprotein (GP), can stimulate pericytes to secrete vascular endothelial growth factor (VEGF) to cause iBRB breakdown. VEGF downregulated tight junction protein claudin-1 between retinal endothelial cells to disrupt iBRB. Moreover, we found that Ebola GP is crucial for VEGF secretion and iBRB breakdown. In vivo, we also found that EBO-VLPs can cause iBRB breakdown through VEGF secretion in rats. This study provides information that is important for understanding the mechanism by which EBOV disrupts the iBRB in persistent infection and provides a new insight for the treatment of EBOV persistence in EVD survivors.

## Results

### EBO-VLPs disrupt the iBRB in the in vitro model

**Fig 1:**
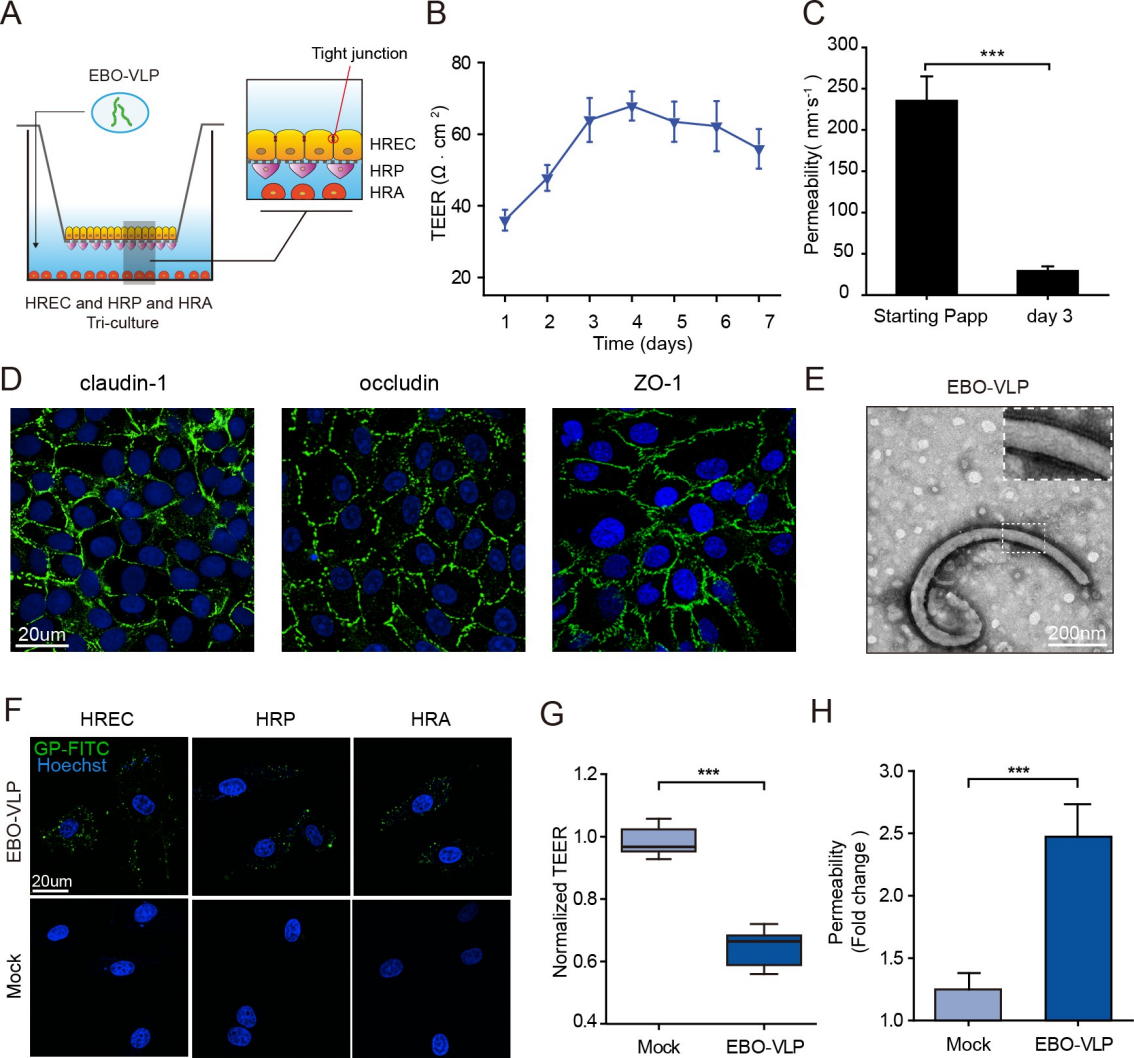
EBO-VLPs disrupt the iBRB in the in vitro model. (A) Schematic overview of the iBRB tri-culture model in a Transwell system of HREC, HRP and HRA. EBO-VLPs were added to the lower chamber of the tri-culture iBRB model, which represents the retinal tissue. (B) Assessment of the integrity of the in vitro barrier models by TEER every day for one week. The results are presented as the means ± standard deviation of six independent experiments. (C) Na-F permeability of iBRB tri-culture models at 6 hours and 3 days post HREC seeding. The results are presented as the means ± standard deviation of six independent experiments. (D) Images of HREC showing the expression of claudin-1, occludin, and ZO-1. Claudin-1, occludin, and ZO-1 are shown in green, and cell nuclei were stained with DAPI (blue). Representative images of three independent experiments are shown. (E) TEM images of EBO-VLPs. (F) Images of HRECs, HRPs and HRAs treated with EBO-VLPs and immunostained with anti-GP antibodies (green). Cell nuclei were stained with DAPI (blue). Representative images of three independent experiments are shown. The fluorescent images were taken with a 60× magnification objective lens under a confocal microscope. (G-H) Integrity of the tri-culture iBRB model after EBO-VLP administration. TEER values (G) and Na-F permeability (H) of the iBRB model were examined at 48 h after EBO-VLP administration. TEER values were normalized to those of iBRB models themselves before EBO-VLP administration. The box and the whisker present the median ± percentiles (25–75%) and range, respectively. The fold change of permeability compared with iBRB model itself before EBO-VLP administration is presented as the mean ± standard deviation. All values were determined in six independent experiments. Statistical analysis was performed using Student’s t test. *** p< 0.001.

In this study, we used a tri-culture Transwell iBRB model to study the effects of EBOV on the iBRB. To establish the iBRB model, human retinal endothelial cells (HRECs) were seeded on the Transwell insert, human retinal pericytes (HRPs) were seeded on the bottom side of the Transwell insert, and primary human retinal astrocytes (HRAs) were grown on the bottom of the culture dish (Fig 1A). We evaluated the barrier properties of this iBRB model by measuring transepithelial electrical resistance (TEER) and endothelial permeability. The results showed that the TEER values reached ~70 ohm∙cm^2^ and stabilized at 3 to 6 days after seeding (Fig 1B). The permeability of Na-F (29.5 ± 5.6 nm∙s^-1^) also verified the integrity of the iBRB at 3 days post HREC seeding compared with 6 hours post seeding (Fig 1C). HRECs in the iBRB model exhibited a classic morphology with the expression of tight junction proteins, claudin-1, occludin and zonula occludens-1 (ZO-1) (Fig 1D).

EBO-VLPs consisting of the matrix protein VP40 and GP were constructed and used to investigate effects on the iBRB in biosafety level 2 condition. EBO-VLPs are useful tools to study the early events of EBOV pathogenesis [25]. EBO-VLPs were characterized by transmission electron microscopy (TEM), which showed a typical filamentous morphology similar to that of wild-type EBOV (Fig 1E). When EBO-VLPs were incubated with HRECs, HRPs or HRAs, EBO-VLPs can enter these cells at 6 hours post incubation (Fig 1F). EBO-VLPs were detected in these cells in a dose-dependent manner, and the percentage of cells infected by EBO-VLPs reached the maximum level at the concentration of 50 μg/mL (S1 Fig). These results showed that HRECs, HRPs and HRAs are all permissive for EBO-VLP uptake.

Then, 50 μg/mL of EBO-VLPs were added to the lower chamber of the tri-culture iBRB model at post 3 days after HREC seeding, which represented retinal tissue (Fig 1A). TEER (S2A Fig) and Na-F permeability (S2B Fig) were measured to evaluate the integrity of the iBRB model at 24 h, 48 h and 72 h post EBO-VLP treatment. A 35% reduction in the TEER was observed at 48 h after EBO-VLP administration compared with that before EBO-VLP administration (Fig 1G). The permeability of Na-F showed a 2.5-fold increase at 48 h after EBO-VLP administration (Fig 1H). 10 ng/mL of TNF-α was used as a positive control which is known to disrupt the barrier [26]. The iBRB model showed a decrease of TEER and increase of Na-F permeability, representing the breakdown of iBRB caused by TNF-α (S2C, S2D Fig). This confirmed that our constructed tri-culture model could reflect the property and its change of iBRB. These data showed that iBRB integrity decreased significantly following EBO-VLP treatment, suggesting that EBO-VLPs caused iBRB breakdown.

### The breakdown of iBRB by EBO-VLP is not attributable to direct cytotoxicity on retinal endothelial cells

**Fig 2:**
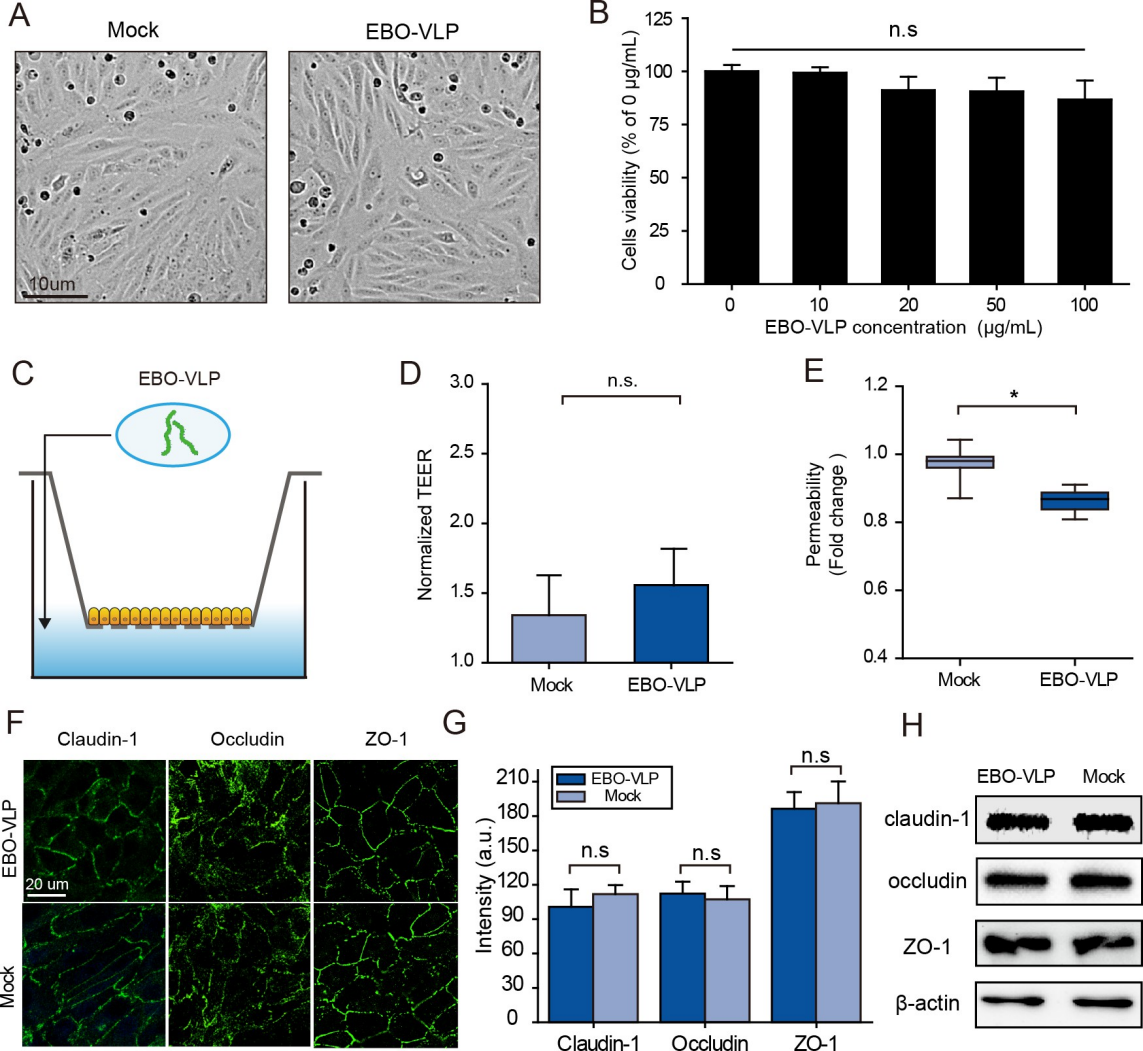
iBRB breakdown by EBO-VLPs is not attributable to direct cytotoxicity on HRECs. (A) Phase contrast images of retinal endothelial cell mono-layers with or without 48 h of EBO-VLP stimulation. Representative images of three independent experiments are shown. (B) Viability of HREC treated with different concentrations of EBO-VLPs. All data were normalized to the mock group. The results are presented as the means ±standard deviation of three independent experiments. (C) Experimental schematic of EBO-VLPs addition to the mono-culture iBRB model. (D-E) Changes in the integrity of the mono-culture iBRB model after EBO-VLP administration. Na-F permeability (D) and the TEER values (E) of the iBRB model were examined 48 h after EBO-VLP administration. TEER values were normalized to those of iBRB models themselves before EBO-VLP administration. The box and the whisker present the median ± percentiles (25–75%) and range, respectively. The fold change of permeability compared with iBRB model itself before EBO-VLP administration is presented as the mean ± standard deviation. All values were determined in six independent experiments. (F) Images of HRECs in the mono-culture iBRB model showing the expression of claudin-1, occludin, ZO-1 (green) and cell nuclei stained with DAPI (blue). Representative images of three independent experiments are shown. The fluorescent images were taken with a 60× magnification objective lens under a confocal microscope. (G) Quantification of the fluorescence intensity of claudin-1, occludin, and ZO-1 after EBO-VLP administration. The regions for fluorescence intensity were determined in four independent experiments. (H) Western blot analysis of claudin-1, occludin and ZO-1 expression in HREC 48 h after EBO-VLP administration in mono-culture iBRB models. Representative images of three independent experiments are shown. Statistical analysis was performed using Student’s t test. *p < 0.05, **p < 0.01.

To explore the mechanism of iBRB breakdown, we examined whether EBO-VLPs caused direct cytotoxicity to endothelial cells. As shown in Fig 2A, the cytopathic effect was not detected in HRECs treated with EBO-VLPs. We then examined the effect of EBO-VLPs on the viability of HRECs. At 48 h after EBO-VLP administration, the viability of HRECs was tested by a CCK-8 assay kit. The results showed that EBO-VLPs did not affect the viability of HRECs (Fig 2B).

Then, we used a mono-culture iBRB model to evaluate the effect of EBO-VLPs on the integrity of the iBRB. HRECs were seeded on the Transwell insert to establish the mono-culture iBRB model (Fig 2C). The barrier was characterized by the TEER value (~50 ohm∙cm^2^) and Na-F permeability (80.5 ± 1.2 nm∙s^-1^) to verify its integrity (S3A and S3B Fig). The tight junction proteins claudin-1, occludin and ZO-1 between HRECs were examined (S3C Fig). After 50 μg/mL of EBO-VLPs were added to the lower chamber of the model (Fig 2C), the endothelial permeability showed no apparent increase during the following 2 days (Fig 2D). The TEER showed only a 14% decrease (Fig 2E), which was much lower than that in the tri-culture model (Fig 1G). Moreover, the distribution patterns and expression levels of tight junction proteins showed no differences in the HRECs mono-culture model with or without EBO-VLPs (Fig 2F 2G and 2H). Thus, EBO-VLPs did not cause iBRB breakdown in the HRECs mono-culture model. And combined with the above results it can be deduced that iBRB breakdown in the tri-culture model is not caused by direct cytotoxicity of EBO-VLPs on retinal endothelial cells.

### EBO-VLP can significantly stimulate pericytes to secrete VEGF

**Fig 3:**
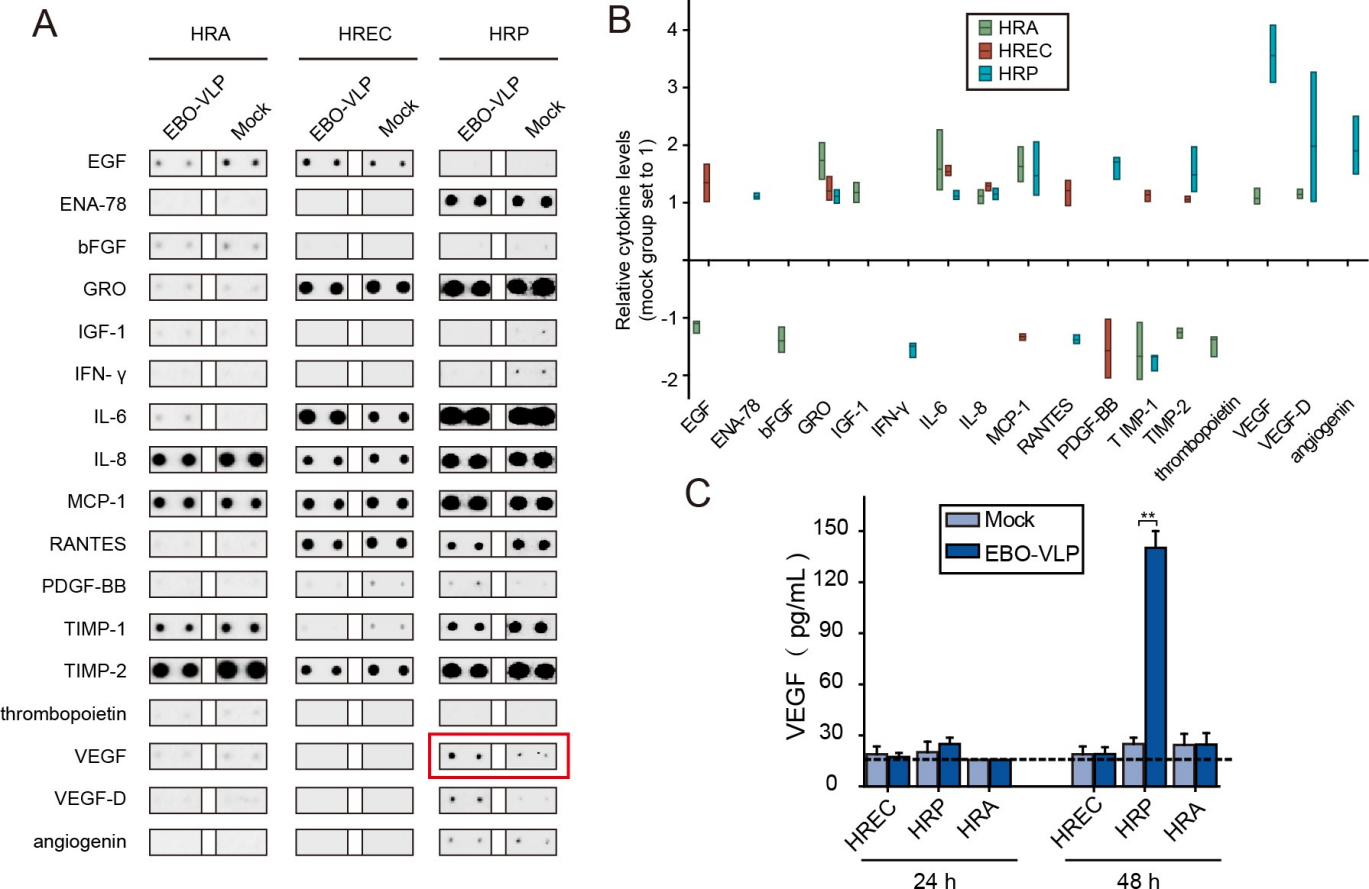
EBO-VLPs can significantly stimulate pericytes to secrete VEGF. (A) Representative cytokines secreted by HREC, HRP and HRA after 48 h of EBO-VLP stimulation. Representative images of three independent experiments are shown. (B) Relative cytokine levels were normalized to the mock group without EBO-VLP administration, which was set as 1. The floating bar plot shows the mean, minimum and maximum levels of each cytokine in three independent experiments. Downregulation of cytokines expression were displayed with negative numbers. (C) Changes in VEGF expression after EBO-VLP administration, as quantified by ELISA. The horizontal dashed line marks the limit of detection of the assay. The results are presented as the means ± standard deviation of three independent experiments. Statistical analysis was performed using Student’s t test. *p < 0.05, **p < 0.01.

Some viruses can trigger the secretion of cytokines or inflammatory factors to cause endothelial dysfunction and iBRB breakdown. To clarify the mechanism of iBRB breakdown caused by EBO-VLPs, we examined cytokine secretion in cells stimulated with EBO-VLPs. As shown in Fig 3A, the candidate cytokines associated with endothelial permeability and barrier function secreted by retinal endothelial cells, pericytes and astrocytes were screened using an antibody array. The screening results showed several cytokines were stimulated in these cells by EBO-VLPs, such as VEGF secreted by pericytes, EGF secreted by endothelial cell, IL-6 secreted by astrocytes, and so on (Fig 3B). Among them, VEGF secretion by pericytes showed the most significant increase (Fig 3B).

ELISA was performed to verify VEGF induction by EBO-VLPs. The results showed that VEGF secretion was highly increased to 140 pg/mL in HRPs at 48 h after EBO-VLP administration, while VEGF levels remained low in HRECs and HRAs (Fig 3C). This finding suggested that EBO-VLPs significantly stimulated pericytes to secrete VEGF. Considering that VEGF is a typical cytokine that causes barrier dysfunction, the secretion of VEGF by pericytes is likely to play an important role in iBRB breakdown by EBO-VLPs.

### EBO-VLPs cause iBRB breakdown by secretion of VEGF in pericytes

**Fig 4:**
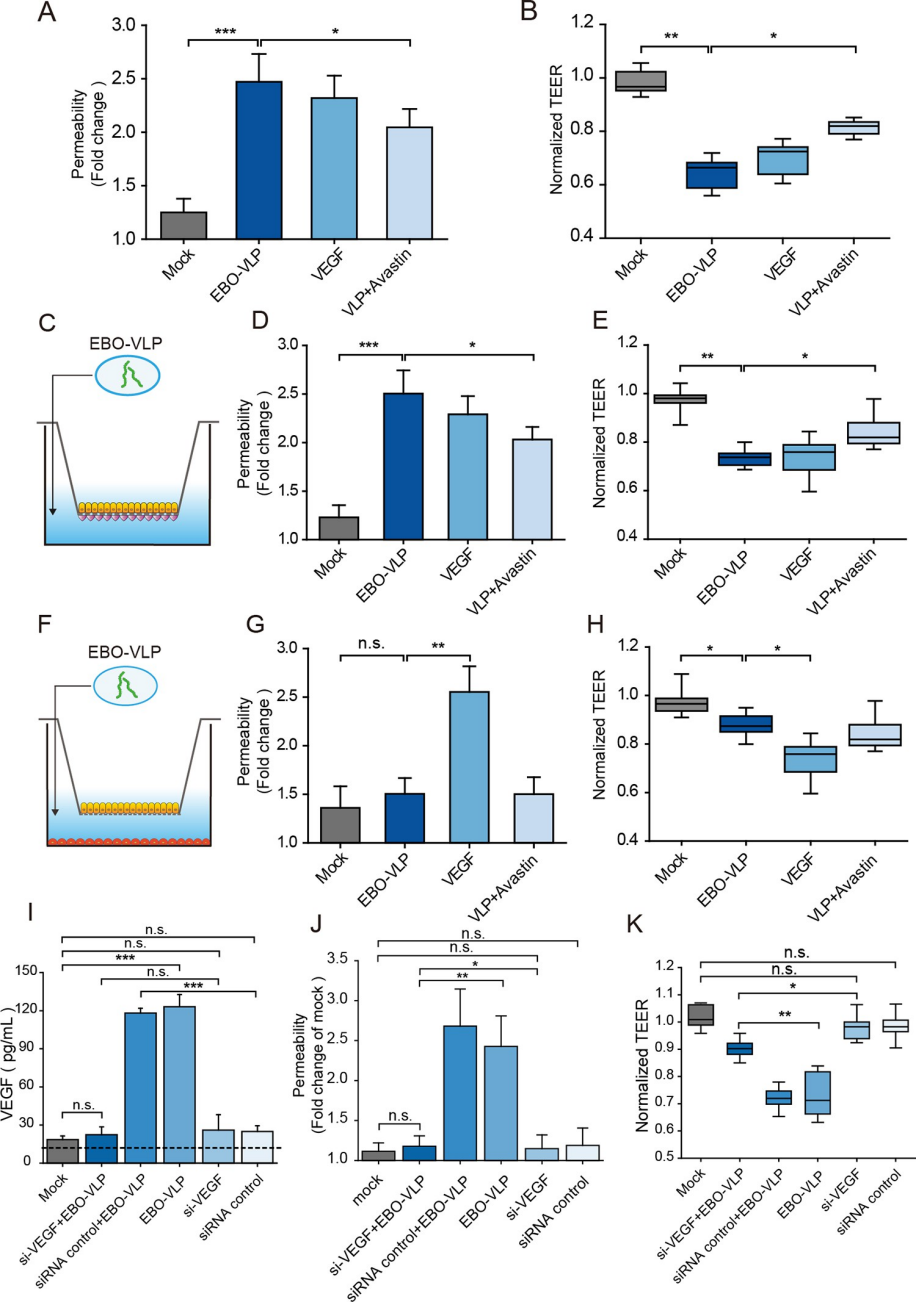
EBO-VLP causes iBRB breakdown through pericytes secretion of VEGF. (A-B) Integrity of the tri-culture iBRB model after EBO-VLP, EBO-VLP + Avastin and VEGF treatment. Na-F permeability (A) and the TEER values (B) of the iBRB model were examined 48 h after EBO-VLP administration. TEER values were normalized to those of iBRB models themselves before EBO-VLP administration. The box and the whisker present the median ± percentiles (25–75%) and range, respectively. The fold change of permeability compared with iBRB model itself before EBO-VLP administration is presented as the mean ± standard deviation. All values were determined in ten independent experiments. (C) Experimental schematic showing the addition of EBO-VLPs to the iBRB co-culture model of HREC and HRP. (D-E) Integrity of the iBRB co-culture (HRECs and HRPs) model after EBO-VLP, EBO-VLP + Avastin and VEGF treatment. Na-F permeability (D) and the TEER values (E) of the iBRB model were examined after 48 h of treatment. TEER values were normalized to those of iBRB models themselves before EBO-VLP administration. The box and the whisker present the median ± percentiles (25–75%) and range, respectively. The fold change of permeability compared with iBRB model itself before EBO-VLP administration is presented as the mean ± standard deviation. All values were determined in ten independent experiments. (F) Experimental schematic showing the addition of EBO-VLPs to the iBRB co-culture model of HREC and HRA. (G-H) Integrity of the iBRB co-culture (HRECs and HRAs) model after EBO-VLP, EBO-VLP + Avastin, and VEGF treatment. Na-F permeability (G) and the TEER values (H) of the iBRB model were examined 48 h after EBO-VLP administration. All values were determined in ten independent experiments. (I) Changes in VEGF expression after EBO-VLP administration, as quantified by ELISA. Pericytes were transfected with si-VEGF or nontargeting control siRNA, followed by treatment with EBO-VLP for 48 h. The horizontal dashed line marks the limit of detection of the assay. The results are presented as the means ± standard deviation of three independent experiments. (J-K) Integrity of the tri-culture iBRB model after EBO-VLP, si-VEGF + EBO-VLP, nontargeting control siRNA + EBO-VLP, and nontargeting control siRNA treatment. Na-F permeability (J) and the TEER values (K) of the iBRB model were examined 48 h after administration. TEER values were normalized to those of iBRB models themselves before EBO-VLP administration. All values were determined in six independent experiments. Statistical analysis was performed using Student’s t test. *p < 0.05, **p < 0.01, ***p < 0.001.

Then, the role of VEGF secretion by pericytes in iBRB breakdown was examined. 10 ng/mL to 100 ng/mL of VEGF were added to the lower chamber of the iBRB tri-culture model, and Na-F permeability (S4A Fig) and TEER (S4B Fig) showed that iBRB breakdown at 50 ng/mL of VEGF at 24 h post VEGF treatment, which was similar to the effect of EBO-VLP treatment (Fig 4A and 4B). In the iBRB model, the VEGF antibody Avastin reduced the effect on iBRB breakdown induced by EBO-VLP (Fig 4A and 4B). The addition of 100 ng/mL of Avastin showed the 54 ± 7.4% of TEER and 41 ± 6.7% of Na-F permeability recovery compared with only EBO-VLP treatment. Whereas, VEGF + Avastin treatment, Avastin alone and control IgG isotype had little effect on iBRB breakdown (S4C and S4D Fig). This result demonstrated that VEGF plays a critical role in iBRB breakdown.

We also used co-culture iBRB models to verify the function of pericytes. The HRECs and HRPs (S5 Fig) or HRAs (S6 Fig) co-culture model were constructed, and iBRB barrier properties were examined. EBO-VLPs were added to the lower chamber of the HRECs and HRPs co-culture model (Fig 4C), and at 48 h post EBO-VLP treatment, the Na-F permeability and TEER were measured. The increase in endothelial permeability (Fig 4D) and the decrease in TEER (Fig 4E) demonstrated that the iBRB was destroyed, which was similar to that in the tri-culture model. The addition of the VEGF antibody Avastin also reduced the effect on VLP-induced iBRB breakdown in the HRECs and HRPs co-culture model (Fig 4D and 4E). However, in the HRECs and HRAs co-culture model, in which there were no pericytes (Fig 4F), EBO-VLP treatment did not affect endothelial permeability or TEER of the iBRB model (Fig 4G and 4H). These data demonstrated that pericytes were essential for secreting VEGF to cause iBRB breakdown.

Furthermore, VEGF KD assay in pericytes was carried out to confirm the role of VEGF in pericytes on iBRB breakdown. Following 48 h treatment with small interfering RNAs (siRNAs), VEGF expression in pericytes was significantly decreased (S7A and S7B Fig), and in VEGF-KD-pericytes VEGF secretion stimulated by EBO-VLP was significantly decreased (Fig 4I). In iBRB model with VEGF-KD-pericytes, EBO-VLP did not cause obvious breakdown of the iBRB (Fig 4J and 4K). VEGF knockdown in pericytes also showed no impact on TEER and Na-F permeability of iBRB (Fig 4J and 4K). The results demonstrated that VEGF knock down in pericytes could alleviate the damage caused by EBO-VLP in iBRB models. Thus, the iBRB breakdown by EBO-VLP was mainly caused by the VEGF secretion in pericytes.

### EBO-VLPs downregulate the tight junction protein claudin-1 in the iBRB

**Fig 5:**
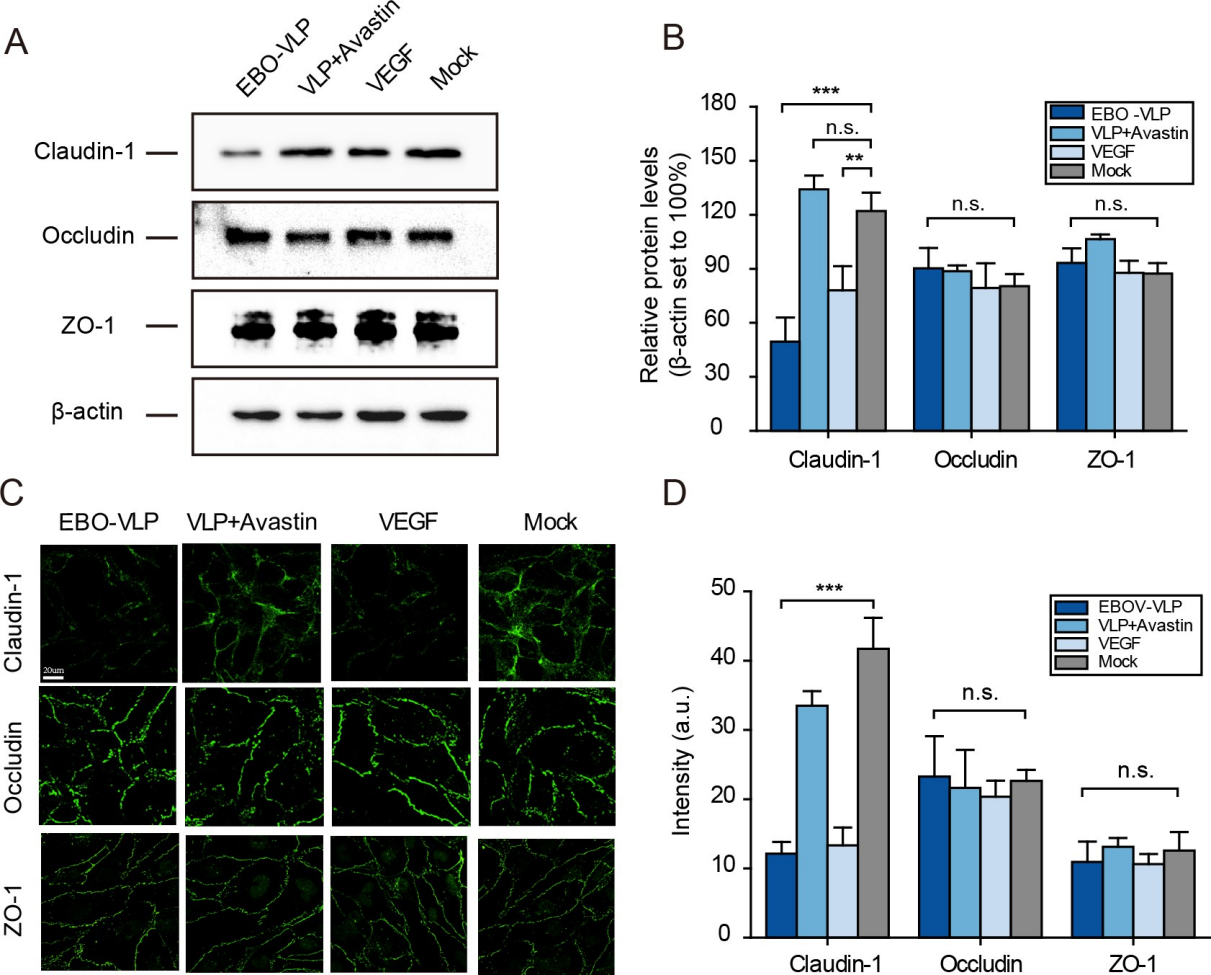
EBO-VLPs downregulate the tight junction protein Claudin-1 in the iBRB. (A) Western blot analysis of claudin-1, occludin and ZO-1 expression in HREC at 48 h in the tri-culture iBRB model. Representative images of three independent experiments are shown. (B) Relative protein levels of claudin-1, occludin and ZO-1 were normalized to β-actin. The results are presented as the means ± standard deviation of three independent experiments. (C) Immunofluorescence images of claudin-1, occludin and ZO-1 expression in HRECs at 48 h post EBO-VLP treatment in the tri-culture iBRB model. Representative images of three independent experiments are shown. (D) Quantification of the fluorescence intensity of claudin-1, occludin and ZO-1 after EBO-VLP administration. The regions for fluorescence intensity analysis were determined in four independent experiments. **p < 0.01, ***p < 0.001.

The destruction of the barrier is often related to the loss of tight junction protein expression. We then examined the effect of EBO-VLPs on tight junction protein expression in the tri-culture iBRB model. Western blot analysis showed that after EBO-VLP administration, the expression of claudin-1 was significantly downregulated, while the expression of occludin and ZO-1 was unchanged (Fig 5A and 5B). When Avastin was added to the system, the expression of claudin-1 was not downregulated compared with that in the EBO-VLP group (Fig 5A and 5B). Immunofluorescence analysis showed discontinuous connections and reduced claudin-1 fluorescence intensity in EBO-VLP-treated HRECs (Fig 5C and 5D). Avastin could retain claudin-1 distribution and expression (Fig 5C and 5D). In contrast, occludin and ZO-1 did not show any differences with or without EBO-VLPs.

Similar to EBO-VLPs, VEGF could also downregulate the tight junction protein claudin-1. As shown in Fig 5B, the amount of claudin-1 was reduced in VEGF-treated HRECs. Immunofluorescence analysis showed that claudin-1 was downregulated from the plasma membrane after VEGF treatment (Fig 5C). In the HRECs and HRPs co-culture model, the downregulation of claudin-1 was also observed following EBO-VLP administration, and this effect could be reduced by Avastin treatment (S8A Fig). In contrast, the downregulation of claudin-1 was not observed in the iBRB mono-culture model or the iBRB co-culture model by HRECs and HRAs, which lacked pericytes (S8B and S8C Fig). These results revealed that EBO-VLPs caused the downregulation of claudin-1 in retinal endothelial cells by stimulating VEGF secretion by pericytes, which could contribute to iBRB breakdown.

### The GP of EBOV plays a major role in iBRB breakdown

**Fig 6:**
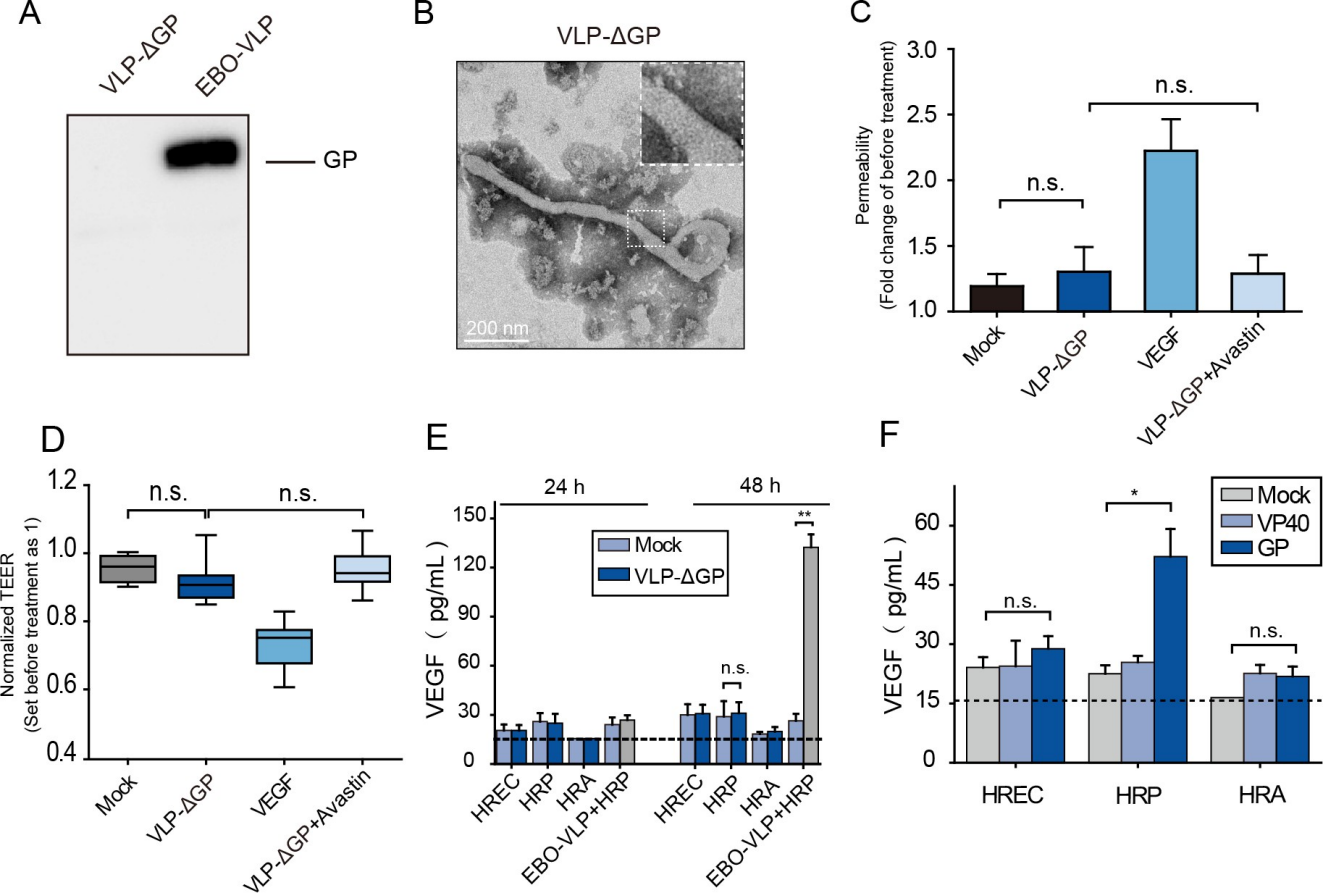
GP plays the major role in the destruction of the iBRB. (A) Western blot analysis of VLP-ΔGP using an anti-GP antibody. Representative images of three independent experiments are shown. (B) TEM image of VLP-ΔGP. (C) Permeability and (D) TEER of the tri-culture iBRB model were measured 48 h after VLP-ΔGP, VLP-ΔGP + Avastin and VEGF treatment. TEER values were normalized to those of iBRB models themselves before EBO-VLP administration. The box and the whisker present the median ± percentiles (25–75%) and range, respectively. The fold change of permeability compared with iBRB model itself before EBO-VLP administration is presented as the mean ± standard deviation. All values were determined in six independent experiments. (E) The expression of VEGF in HRP after VLP-ΔGP treatment, as quantified by ELISA. The horizontal dashed line marks the limit of detection of the assay. The results are presented as the means ± standard deviation of three independent experiments. (F) The expression of VEGF in HRP after EBOV VP40 and GP was expressed in HRPs. The horizontal dashed line marks the limit of detection of the assay. The results are presented as the means ± standard deviation of three independent experiments. Statistical analysis was performed using Student’s t test. *p < 0.05, **p < 0.01.

EBO-VLPs without GP (VLP-ΔGP) were also constructed and examined in iBRB models. VLP-ΔGP consisting of VP40 exhibited filamentous morphology without spike structures (Fig 6A and 6B). When 50 μg/mL of VLP-ΔGP was added to the iBRB model, endothelial permeability (Fig 6C) and TEER (Fig 6D) showed no obvious changes; thus, the iBRB was not affected by VLP-ΔGP. This result was compared with the iBRB disruption caused by EBO-VLPs consisting of VP40 and GP and showed that GP plays a key role in iBRB breakdown. Then, HRECs, HRPs and HRAs were treated with VLP-ΔGP to examine the VEGF secretion. The results showed that 50 μg/mL of VLP-ΔGP did not stimulate VEGF secretion by pericytes in 48 h post VLP-ΔGP treatment (Fig 6E). This finding demonstrated that GP was important for the stimulation of VEGF. Furthermore, EBOV VP40 and GP were expressed in HRPs, and the secretion of VEGF was measured. The results showed that in HRPs with GP, VEGF was highly secreted, while in HRPs with VP40, VEGF secretion remained at the same level as that in the control (Fig 6F). These results demonstrated that GP protein plays a major role in iBRB breakdown.

### EBO-VLPs cause the destruction of the iBRB in vivo

**Fig 7:**
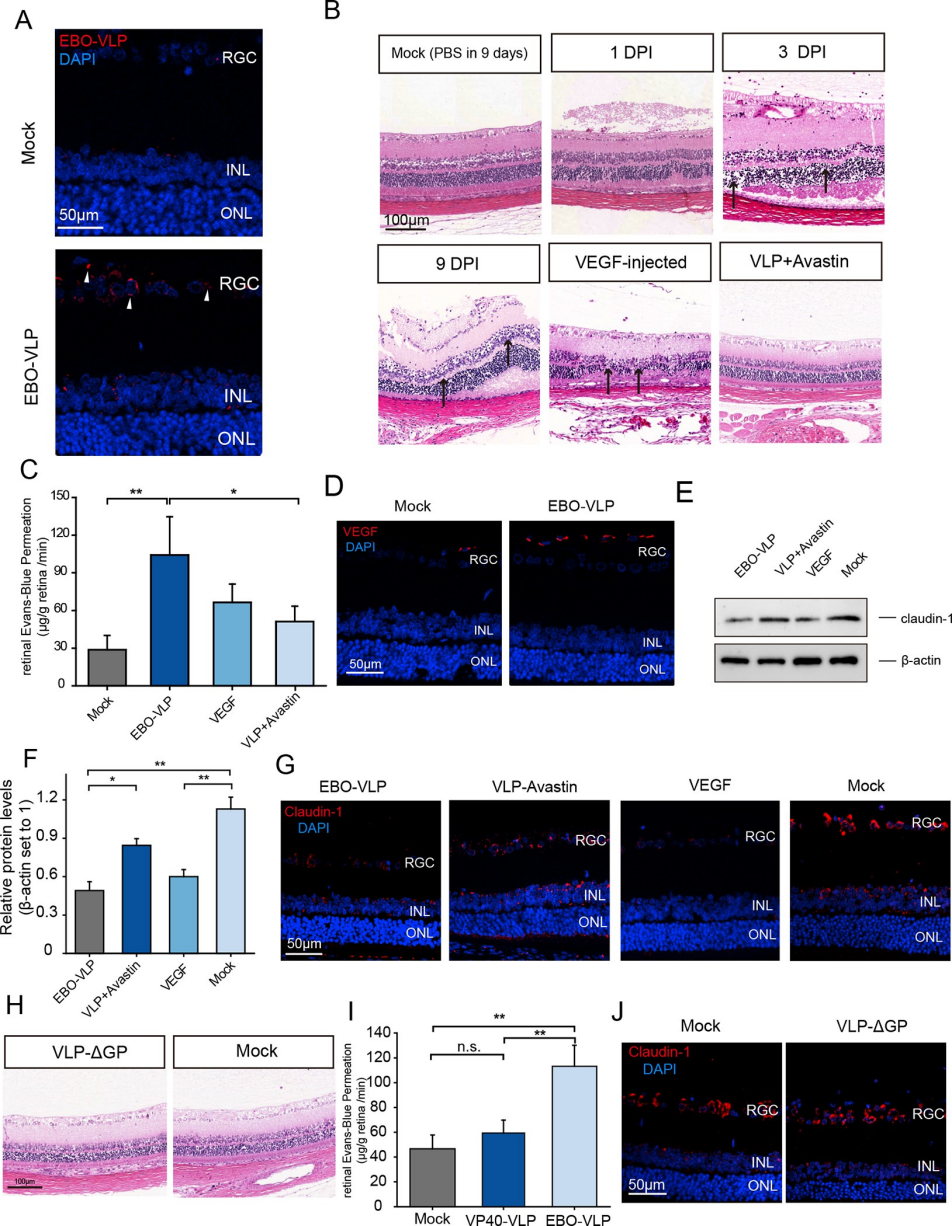
EBO-VLPs damage the iBRB in vivo. (A) Immunohistofluorescence analysis of EBO-VLPs in the retinal tissue using anti-GP antibodies (red). The white arrow indicates EBO-VLPs. Representative images of three independent experiments are shown. The fluorescent images were taken with a 60× magnification objective lens under a confocal microscope. (B) H&E staining of retinas from rats treated with Avastin and/or EBO-VLPs and VEGF. Black arrows indicate the pathological destruction of the retina. Magnification: ×20. Representative images of three independent experiments are shown. (C) Evans blue assay showing rat retinal permeability at 2 days after EBO-VLP injection. The results are presented as the means ± standard deviation of four independent experiments. (D) Immunohistofluorescent staining of retinal sections was performed to analyze VEGF (red). Nuclear staining with DAPI shows the retinal layers. Representative images of three independent experiments are shown. (E) Western blot analysis of claudin-1 expression in rat retinas, which was normalized against β-actin. Representative images of three independent experiments are shown. (F) Relative protein levels of claudin-1 were normalized to β-actin. The results are presented as the means ± standard deviation of three independent experiments. (G) Immunohistofluorescent staining of claudin-1 in retinal sections. Representative images of three independent experiments are shown. (H) H&E staining of retinas from rats treated with or without VLP-ΔGP. Magnification: ×20. Representative images of three independent experiments are shown. (I) Evans blue assay showing rat retinal permeability at 2 days after VLP-ΔGP injection. The results are presented as the means ± standard deviation of four independent experiments. (J) Images of immunofluorescence staining of claudin-1 in retinal sections treated with VLP-ΔGP. Representative images of three independent experiments are shown. Statistical analysis was performed using Student’s t test. *p < 0.05, **p < 0.01.

Next, we further examined iBRB breakdown caused by EBO-VLPs in vivo. EBO-VLPs were delivered into the vitreous bodies of rats by intravitreal injection. Two days later, immunohistofluorescence analysis was performed to examine EBO-VLPs distribution. We found that EBO-VLPs were distributed mainly in the ganglion cell layer (GCL) and inner nuclear layer (INL) of the retina (Fig 7A). Morphological damage to the retina was detected at 1 d, 3 d, and 9 d post injection. As shown in Fig 7B, gradual pathological damage was observed in the INL and the outer nuclear layer (ONL) of the retina following EBO-VLP administration compared with no treatment control. Evans blue dye was also used to evaluate the permeability of the iBRB in rats. The results showed that the permeability of the iBRB was significantly increased after EBO-VLP administration at 9 days post injection (Fig 7C), which indicated that EBO-VLPs caused iBRB breakdown in rats.

After EBO-VLPs injection, the expression of VEGF was measured in the GCL by immunohistofluorescence analysis. VEGF was highly increased in the ganglion cell layer, where EBO-VLPs were distributed at 9 days post injection (Fig 7D). VEGF was also injected into the vitreous body by intravitreal injection, and it caused morphological damage and increased iBRB permeability at 9 days post injection (Fig 7B and 7C). The results verified that VEGF played an important role in iBRB damage in vivo. Claudin-1 protein levels were measured in the retinas of rats after EBO-VLP injection. As shown in Fig 7E and Fig 7F, western blot and immunofluorescence assays showed that the expression of claudin-1 in the retina was significantly decreased 9 days after EBO-VLP administration.

In rats injected with EBO-VLPs, the VEGF antibody Avastin was also delivered to the retina by intravitreal injection. Avastin inhibited the disorganization of the INL and ONL of the retina induced by EBO-VLPs at 9 days post EBO-VLP administration (Fig 7B). Avastin treatment alleviated the increase in iBRB permeability compared with EBO-VLP-treated rats. (Fig 7C), and the expression of claudin-1 showed no downregulation (Fig 7E and 7F). Thus, in rats, inhibiting VEGF reduces the effect on iBRB breakdown caused by EBO-VLPs. VLP-ΔGP was also injected into the vitreous body. VLP-ΔGP was distributed in the GCL and INL of the retina (S9B Fig) at 2 days post injection. Morphological changes in the retina were not observed after VLP-ΔGP administration at 9 days post injection (Fig 7H). iBRB permeability showed no difference in VLP-ΔGP-treated and untreated rats (Fig 7I). The expression of claudin-1 in the retina was also not affected by VLP-ΔGP (Fig 7J). These results verified the key role of EBOV GP in iBRB breakdown in rats.

## Discussion

EBOV persistence in the eyes can cause ocular complications and even EVD recurrence, which shows that EBOV in retinal tissue can break through the iBRB to enter the bloodstream. To date, the mechanism by which EBOV affects the iBRB remains unclear. Here, by using the in vitro iBRB model we found that EBO-VLPs could disrupt the iBRB, but they did not cause direct cytotoxicity to retinal endothelial cells. Cytokine screening revealed that VEGF secretion by pericytes was stimulated by EBO-VLPs and caused iBRB breakdown. VEGF downregulated claudin-1 to destroy the iBRB, and Ebola GP was crucial in this process. Furthermore, this mechanism was also verified in rats. Our findings provide an important understanding of the virus-iBRB interaction during EBOV persistence.

The BRB isolates retinal tissue from vessels to maintain the specialized environment of the retina [27]. In our study, we constructed an in vitro iBRB model using a Transwell system, which separated the two spaces with a permeable membrane to distinguish the retinal tissue layer from the vascular layer. Transwell tri-culture and co-culture models have been proven to be correct and valuable tools to study the physiology and pathology of blood-retinal-barrier. By these barrier models, TEER and permeability assay can be used to quantitatively measure the integrity of the barrier [28,29]. This model allowed us to explicitly add EBO-VLP to the space where the retinal tissue is represented, so as to study the mechanism of iBRB breakdown caused by EBOV in retinal tissue under persistent infection. We observed that after EBO-VLP administration, the integrity of the iBRB was decreased, and the permeability of endothelial cells was significantly increased. This result clearly showed that EBO-VLPs caused iBRB breakdown. According to previous reports, EBOV persistence leads to ocular symptoms and the infiltration and accumulation of inflammatory cells in the retina [10,14,27]. iBRB breakdown can contribute to ocular complications [15,17,30]. EBOV can generate defective interfering particles (DIPs) during infection, which may play a role in persistent infection [31]. EBO-VLP-induced iBRB breakdown provides a new clue to the pathogenesis of DIPs. iBRB breakdown also suggests that EBOV can spread from the retina to the blood and cause EVD recurrence. Understanding iBRB breakdown is important for the treatment of EBOV persistence.

Viruses may damage the iBRB in two ways. One is to directly cause cytopathic effects on endothelial cells in the barrier [32,33]. The other way is to hijack cytokines or inflammatory factors and indirectly cause endothelial dysfunction [34]. For example, Japanese encephalitis virus (JEV) infects mast cells and causes them to secrete chymase to promote blood–brain barrier (BBB) breakdown and CNS infection [24]. SARS-CoV-2 can increase the levels of IL-6, TNF-α and MCP1 in the brain to disrupt the BBB [23]. Here, we found that EBO-VLPs stimulate pericytes to secrete VEGF to disrupt the iBRB. VEGF is generally believed to increase the permeability of blood vessels [35]. It often plays important roles in inflammation and pathological conditions [36]. Many ocular diseases associated with blindness are related to VEGF, including diabetic retinopathy and neovascular age-related macular degeneration [16]. Some other viruses also hijack VEGF to increase barrier leakage. Andes virus can stimulate VEGF secretion by human lung endothelial cells and disrupt the endothelial cell barrier [37]. HSV-1 can increase the production of VEGF in astrocytes to disrupt the BBB [38]. Our finding that EBO-VLPs induced VEGF secretion by pericytes revealed a new mechanism of iBRB breakdown. Furthermore, we showed that Avastin, a VEGF neutralizing antibody, could reduce barrier dysfunction induced by EBO-VLPs administration. It is consistent with previous study that the barrier dysfunction of bovine retinal endothelial cells caused by VEGF could be restored by inhibiting VEGF [39,40]. Ocular anti-VEGF therapy has been shown to be an effective treatment for some retinal diseases [39]. Our data also provide a theoretical basis for the treatment of iBRB dysfunction and its related ocular complications in Ebola survivors.

We found that claudin-1 was downregulated by EBO-VLP-induced VEGF. Tight junction proteins such as occludin, claudin-1 and ZO-1 play key roles in restricting paracellular permeability to establish the endothelial barrier [24,32,41]. Some viruses can cause the degradation or dissociation of tight junction proteins to disrupt the barrier. For example, JEV causes the degradation of ZO-1 and claudin-5 to disrupt the BBB [24]. Zika infection decreases the expression of ZO-1 and occludin in human placental trophoblasts in the blood-placenta barrier [42]. It has also been reported that reduced expression of claudin-1 in bovine retinal endothelial cells leads to low integrity of the iBRB [39]. Here, the downregulation of claudin-1 provides further explanation for iBRB breakdown caused by EBOV and VEGF.

Our results showed that Ebola GP was crucial for VEGF stimulation and iBRB breakdown. GP is the only transmembrane protein on the surface of EBOV particles and has a variety of functions in addition to binding to receptors to facilitate vial entry [2,4,14]. GP is the major EBOV immunogen that stimulates the host immune response [43]. GP is also the main determinant of cytotoxicity and cell activation [44,45]. Ebola GP stimulates TNF-α secretion to trigger T lymphocyte death [46]. GP in VLPs can also activate macrophages to produce high levels of proinflammatory cytokines, including TNF-α, IL-6 and IL-8, causing cytokine storms [25]. Our results identify a new function of Ebola GP, which participates in VEGF stimulation and iBRB breakdown. This function provides new insight into EBOV pathogenesis.

**Fig 8:**
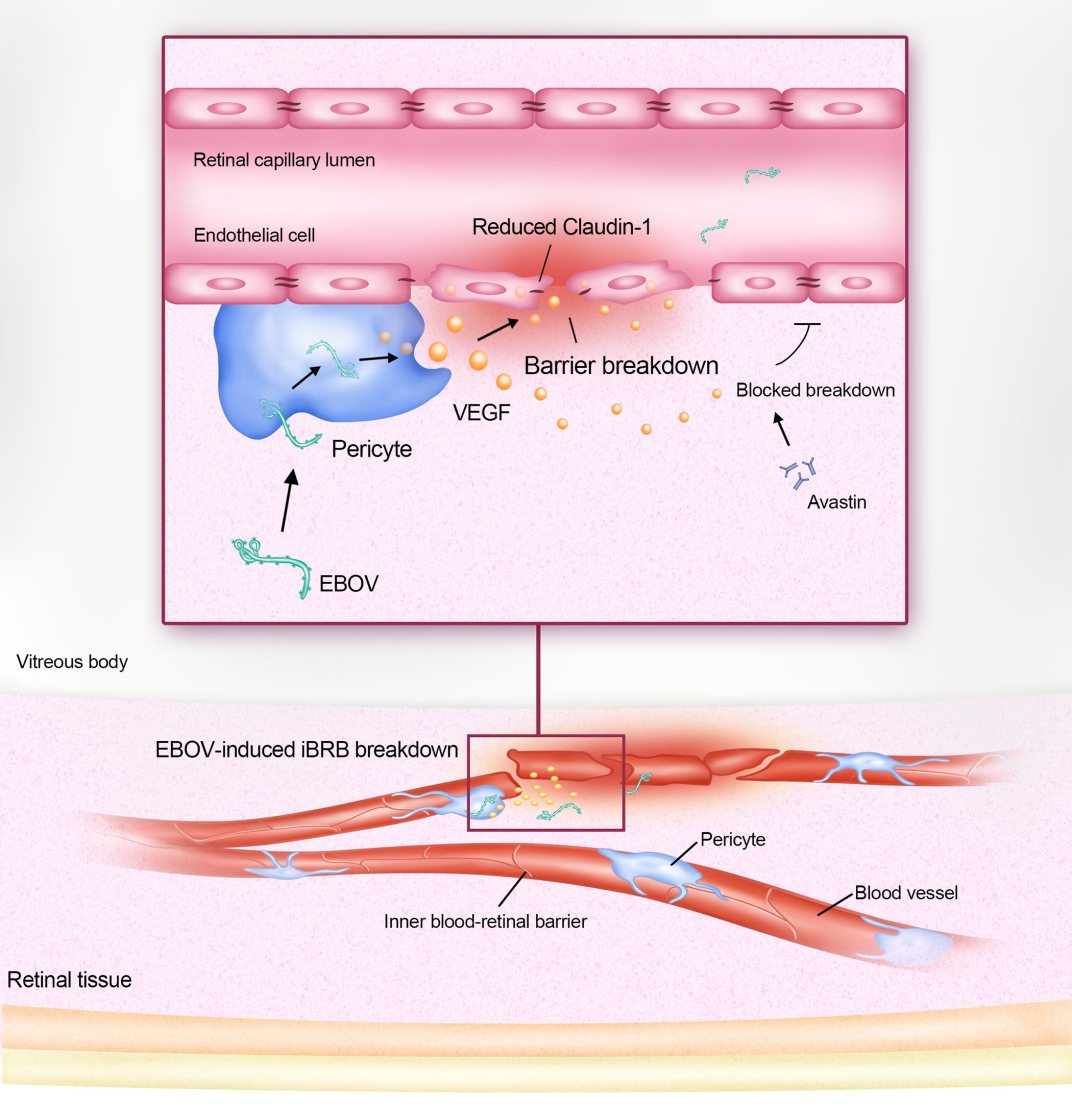
Model for the iBRB breakdown affected by EBOV. EBOV stimulates pericytes to secrete VEGF to cause the iBRB breakdown. VEGF disrupts iBRB through downregulation of the tight junction protein claudin-1. The VEGF antibody Avastin can reduce the effect on VLP-induced iBRB breakdown.

In summary, we revealed a novel mechanism by which EBOV affects the iBRB (Fig 8). We found that EBO-VLPs cause iBRB breakdown by stimulating pericytes to secrete VEGF. VEGF causes the downregulation of the tight junction protein claudin-1 to disrupt the iBRB. Ebola GP plays a key role in VEGF stimulation and iBRB breakdown. These findings provide mechanistic insights into that EBOV breaks through the iBRB during EBOV persistence in retinal tissue, which will assist in developing new strategies to treat EBOV persistence in EVD survivors.

## Materials and methods

### Ethics statement

All animal experiments were reviewed and approved by the Animal Care Committee of Wuhan Institute of Virology (Permit number: WIVA18201901) and were performed in accordance with the animal ethics guidelines of the Chinese National Health and Medical Research.

### Cell culture

Immortalized HRECs (Zhong Qiao Xin Zhou, Shanghai, China) were maintained in endothelial cell medium (ECM, ScienCell, San Diego, California, USA) containing 5% fetal bovine serum (FBS, Thermo Fisher), 1% endothelial cell growth supplement (ECGS), 100 U/mL penicillin and 0.1 mg/mL streptomycin. Immortalized HRPs (Zhong Qiao Xin Zhou) were grown in Dulbecco’s modified Eagle’s medium (DMEM) containing 10% FBS, 100 U/mL penicillin, and 0.1 mg/mL streptomycin. Primary HRAs (ScienCell) were cultured in astrocyte medium (AM, ScienCell) with 5% FBS, 1% astrocytes growth supplement (AGS), 100 U/mL penicillin, and 0.1 mg/mL streptomycin.293T cells (ATCC, ACS-4500) were maintained in DMEM containing 10% FBS, 100 U/mL penicillin, and 0.1 mg/mL streptomycin. All cells were cultured at 37°C and 5% CO_2_ in a humidified incubator.

### Plasmids and antibodies

EBOV VP40 and GP were engineered into pcDNA3.1+ to produce EBO-VLPs in 293T cells. The following antibodies were used in this study: mouse anti-EBO-GP IgG (our laboratory), mouse rabbit claudin-1 antibody (Abcam, ab211737), rabbit occludin antibody (Abcam, ab216327), anti-ZO-1 (Thermo Fisher, 1A12), rabbit VEGF antibody (Beyotime, AV202), and Alexa Fluor 488-and555-labeled goat anti-mouse and anti-rabbit IgG (Cell Signaling Technology).

### Production and purification of VLPs

To construct EBO-VLPs, the pcDNA3.1+-EBO-VP40 and EBO-GP plasmids were cotransfected into 293T cells grown in 10 cm dishes with 54 μg of total DNA at a ratio of 2:1. After 2 days, the supernatants were collected by ultracentrifugation at 25,000 rpm and 4°C for 2.5 h. The precipitate was resuspended in 200 μL of TNE buffer (10 mMTris, 100 mMNaCl, and 1 mM EDTA) and dialyzed by 1L of TNE buffer overnight at 4°C. Dialysis was performed with 8–14 KDMWCO membranes (ShanghaiYuanye Bio-Technology, China).

### Immunofluorescence assay and western blotting

For immunofluorescence analysis, cells and EBO-VLPs were fixed with 4% paraformaldehyde for 20 min at room temperature, permeabilized for 15 min with 0.1% Triton X-100 and blocked with 10% FBS for 1 h. Then, the samples were incubated with antibodies at 4°C overnight. The samples were washed three times with 0.1% Tween 20 in PBS and incubated with the corresponding fluorescent secondary antibodies for 1 h at 37°C. After the samples were washed with PBS containing 0.1% Tween 20 three times, Hoechst 33342 was used to stain the nuclei for 5 min. Images were observed with a Nikon TiE inverted microscope equipped with a 60×, 1.49 NA, oil immersion objective lens (Nikon).

For western blotting, protein concentrations were determined by a Bradford protein assay kit (Beyotime, Shanghai, China). Equal amounts of protein were separated by 12% SDS–PAGE and transferred to PVDF membranes. Then, the membrane was blocked with 5% nonfat milk for 1 h at room temperature and incubated with primary antibodies overnight at 4°C. After being washed with 0.2% PBST 3 times, the membrane was incubated with horseradish peroxidase-conjugated goat anti-mouse or rabbit IgG at 37°C for 1 h. The blots were imaged with a ChemiDoc imaging system (Bio-Rad, USA) by chemiluminescence.

### Transmission electron microscopy analysis

To characterize the shape and size of EBO-VLPs, EBO-VLPs were absorbed onto carbon-coated copper grids for 5 min and negatively stained with 2.0% phosphotungstic acid (PTA, pH 7.0) for 2 min. The samples were analyzed by TEM using a 200 kV HITACHI-7000FA transmission electron microscope.

### Cytokine screening assay and VEGF ELISA

HRECs, HRPs, and HRAs were seeded in 12-well plates. Each well was treated with 50 μg/mL of EBO-VLPs in 2% FBS. The supernatants were collected after 2 days and centrifuged at 10,000 rpm at 4°C for 10 min. Cytokine screening was performed using a human angiogenesis antibody array (Abcam, ab134000). In brief, angiogenesis antibody array membranes were incubated with the supernatants collected after EBO-VLP treatment for 1.5 h at room temperature. After washing, they were incubated with biotinylated antibody cocktail (C1) for 1.5 h hours at room temperature. Then, HRP-conjugated streptavidin were added into each well and incubate for 2 hours at room temperature. The membrane was imaged with a ChemiDoc imaging system (Bio-Rad, USA) by chemiluminescence.

The amount of VEGF in the supernatant was measured by a Human VEGF ELISA Kit (Boster, EK0539). In brief, samples were added to a precoated 96-well plate and incubated for 90 min. Then the plate was incubated with Biotin-labeled anti-human VEGF and Avidin-peroxidase complex according to the manufacturer instruction. The absorption (OD: 450 nm) was quantified using an Enspire Multimode Plate Reader (Massachusetts, USA).

### iBRB model set up

To establish the tri-culture iBRB model, 12-well Transwell inserts (surface area: 1.12 cm^2^; pore size: 0.4 μm; Corning Costar) were coated with collagen type IV (Sigma, C6745) for 6 h at room temperature. Pericytes were seeded (5 × 10^4^ cells/cm^2^) on the bottom side of inserts, which were placed upside down. After 4 h of incubation at 37°C and 5% CO_2_, the inserts were inverted and cultured in 12-well plates. On the same day, astrocytes were plated in a 12-well plate at 7.5 × 10^4^ cells/cm^2^. The next day, the inserts containing pericytes were placed in 12-well plates containing astrocytes, while endothelial cells (10^5^ cells/cm^2^) were seeded on the Transwell inserts with pericytes on the opposite side. Co-culture and mono-culture iBRB models were established by the step in front with only the cells it contained. The Transwell inserts were cultured at 37°C and 5% CO_2_ in a humidified incubator, and the medium was changed every 2 days. The integrity of the in vitro iBRB model was evaluated by the TEER value every day. And the Na-F permeability was measured at 6 h and 3 d post HREC seeding to characterize the integrity of iBRB models.

### iBRB integrity assessment

Barrier integrity was evaluated by TEER measurements using a Millicel-Electrical Resistance System (ERS2) (Millipore Corp, USA). Final TEER values are shown as ohm∙cm^2^ and were calculated by subtracting the TEER values of coated cell-free filters. TEER values were displayed by the average from three measurements.

To evaluate the paracellular permeability of the in vitro BRB model, the movement of Na-F across the in vitro iBRB model from the apical side to the basolateral side was measured. Medium in the upper compartment was replaced by an equal volume of medium containing 10 μg/mL Na-F. After 15 min, the medium in the lower chamber was collected, and the fluorescence (Na-F: excitation 480 nm, emission 535 nm) was quantified using an Enspire Multimode Plate Reader (Massachusetts, USA). The Na-F permeability (P) was calculated as:

$$P=\frac{1}{C_{0}S}\frac{dQ}{dx}$$

C_0_ is the Na-F concentration, S is the total surface area of the transwell membrane, and $\frac{dQ}{dx}$ is the transport rate calculated asthe mass over time.

### The treatment of iBRB models

To evaluate the effect of EBO-VLP and other samples on iBRB models, the in vitro iBRB models were washed and incubated by DMEM with 2% FBS at post 3 days after HREC seeding. iBRB models were measured by TEER and Na-F permeability before treatment to confirm their barrier property. 50μg/mL of EOB-VLP, 50 ng/mL of VEGF and 50μg/mL of EOB-VLP with 100 ng/mL of Avastin (HY-P9906, MCE) were added to the Transwell lower chamber of iBRB models. The TEER and Na-F permeability were measured at the corresponding time point. The TEER was normalized with the value before treatment to reflect the change of barrier integrity after treatment. Na-F permeability showed the fold change after treatment compared with the value before treatment.

### siRNA knockdown assay and qRT-PCR assay

Pericytes were transiently transfected using the Lipofectamine RNAiMax protocol (Life Technologies) with 10 pM siRNAs, according to the manufacturer instruction. The sequence of the siRNA to VEGF is: siVEGF, 5’-GGAGUACCCUGAUGAGAUCdTdT-3’ [47]. After 48 h, cells were harvested for western blot analysis, qRT-PCR or infected with EBO-VLP at the concentration of 50 μg/mL. The secretion of VEGF in the supernatant was measured by Human VEGF ELISA Kit (Boster, EK0539) at 24h and 48h post infection.

For qRT-PCR, total RNA was extracted from infected cells using an Omega HP total RNA isolation kit (Omega Bio-Tek, Inc.). QRT-PCR was carried out using a HiScript II one-step qRT-PCR SYBR green kit (Vazyme) on a Bio-Rad CFX96 real-time PCR system (Bio-Rad Laboratories, Inc). The following primers were used: VEGF-F, 5’-AATGCTTTCTCCGCTCTGAA; VEGF-R, 5’-GCTTCCTACAGCACAGCAGA-3’ [48]. Data analysis was based on the relative quantitation method (2^−ΔΔCt^) to determine relative fold changes. All data were normalized to GAPDH expression levels. Each experiment was performed in triplicate and repeated at least three times independently.

For the VEGF-KD iBRB models, pericytes at the bottom side of the inserts were transiently transfected with 10 pM si-VEGFs or nontargeting siRNAs using the Lipofectamine RNAiMax protocol (Life Technologies). 24 h after siRNA transfection, 50 μg/mL of EBO-VLPs were added to the lower chamber of Transwell insert. The TEER and Na-F permeability were measured in 48 h post EBO-VLP treatment.

### Vitreous injection in rats

Healthy Wistar rats aged 4 weeks were purchased from Beijing Vital River Laboratory Animal Technology Company. After anaesthetization, one drop of 0.5% lidocaine hydrochloride (Sigma–Aldrich, USA) was added to the eye, and the superior nasal region of the eye was exposed. A 33 gauge needle was used to pierce the sclera at a low angle between 10 and 20 degrees, and 2.5 μg/μL of EBO-VLPs or VLP-ΔGP, 50 ng/μL of VEGF or 100 ng/μL of Avastin dissolved in 20μL PBS were injected into the vitreous. Retina tissues were harvested for H&E staining, Western blot or immunofluorescenceanalysis. H&E staining was measured at 1 d, 3 d and 9 d post EBO-VLP injection. Retina tissues of rats with VEGF or EBO-VLP + Avastin treatments were harvested at 9 d post injection for H&E staining. Retina proteins were harvested in RIPA Lysis Buffer (Beyotime,Shanghai, China) for western blot and determined by Bradford protein assay kit.

### Rat iBRB permeability assessment

Evans blue dye was used to examine the permeability of the BRB in vivo. After anaesthetization, Evans blue dye was injected into the tail vein (45 mg/kg; Fisher Scientific). After 2 h, the rats were further perfused with PBS to completely remove the Evans blue dye in the vessels. The retinas were carefully dissected, and the weight was determined after thorough drying. Next, the retinas were incubated in 120μL of formamide for 18 h at 70°C to extract the Evans blue dye. The extract was centrifuged twice at 10,000×g for 1 h at 4°C, and the absorbance was measured at 620 nm. The concentration of Evans blue dye in the extracts was calculated using a standard curve of Evans blue dye in formamide and then normalized to the dried retinal weight.

### Statistical analysis

The data are expressed as the means ± standard deviation (SD). The significance of differences between groups was evaluated by Student`s t test, and P < 0.05 was considered statistically significant.

## Supporting information

S1 Data Excel spreadsheet containing, in separate sheets, the underlying numerical data and statistical analysis for Figure panels 1B, 1C, 1G, 1H, 2B, 2D, 2E, 2G, 3B, 3C, 4A, 4B, 4D, 4E, 4G, 4H, 4I, 4J, 4K, 5B, 5D, 6C, 6D, 6E, 6F, 7C, 7F and 7I. (XLSX)

S1 Fig Quantitation of the percentage of infected cells with different concentration of EBO-VLP treatment. **Related to Fig 1.** (A-C) Percentage of infected cells including HRECs (A), HRPs (B) and HRA (C) with 0, 10, 20, 50 and 100μg/mL EBO-VLP treatment, respectively. The results are presented as the means ± standard deviation of three independent experiments. Statistical analysis was performed using Student’s t-test. All statistical analysis were compared with the 0μg/mL of EBO-VLP treatment, respectively. *p < 0.05, **p < 0.01, ***p < 0.001.
(TIF)

S2 Fig EBO-VLP caused tri-culture iBRB models at 48 h post administration. **Related to Fig 1.** (A-B) Integrity of the tri-culture iBRB model at 0 h, 24 h and 48 h post EBO-VLP administration. TEER values (A) and Na-F permeability (B) of the iBRB model were examined at 0 h, 24 h and 48 h after EBO-VLP administration. The box and the whisker present the median ± percentiles (25–75%) and range, respectively. The fold change of permeability compared with that of iBRB model itself before EBO-VLP administration is presented as the mean ± standard deviation. All values were determined in six independent experiments. (C-D) Integrity of the tri-culture iBRB model at 24 h post 10 ng/mL of TNF-α treatment. TEER values were normalized to those of iBRB models themselves before EBO-VLP administration. The fold change of permeability compared with that of iBRB model itself before EBO-VLP administration is presented as the mean ± standard deviation. All values were determined in six independent experiments. Statistical analysis was performed using Student’s t test. *p < 0.05, **p < 0.01, ***p < 0.001.
(TIF)

S3 Fig Characterization of iBRB mono-culture models. **Related to Fig 2.** (A) Assessment of integrity of mono-culture with HRECs in vitro barrier models by TEER every day in a week. The results are presented as the means ± standard deviation of six independent experiments. (B) Na-F permeability of iBRB mono-culture models at 6 hours and 3 days post HREC seeding. The results are presented as the means ± standard deviation of four independent experiments. Statistical analysis was performed using Student’s t-test. (C) Images of HRECs showing expression of claudin-1, occludin, ZO-1 in mono-culture with HRECs. Claudin-1, occludin, ZO-1 are shown in green and cell nuclei stained with DAPI (blue). Representative images of three independent experiments are shown. The fluorescent Images were taken at 60× magnification objective lens under a confocal microscope. Statistical analysis was performed using Student’s t-test. ***p < 0.001.
(TIF)

S4 Fig The effect of VEGF and Avastin treatments on tri-culture iBRB models. **Related to Fig 4.** (A-B) Integrity of the tri-culture iBRB model at different concentration of VEGF at 0,10, 50 and 100 ng/mL at 24 h. Na-F permeability (A) and TEER values (B) of the iBRB model were examined after VEGF administration. TEER values were normalized to those of iBRB models themselves before VEGF administration. The box and the whisker present the median ± percentiles (25–75%) and range, respectively. The fold change of permeability compared with that of iBRB model itself before EBO-VLP administration is presented as the mean ± standard deviation. All values were determined in six independent experiments. (C-D) Integrity of the tri-culture iBRB model treated with 50 ng/mL of VEGF, 100 ng/ml Avastin, 100 ng/mL of IgG isotype and 50 ng/mL VEGF + 100 ng/ml Avastin in 48 h. TEER values were normalized to those of iBRB models themselves before administration. The box and the whisker present the median ± percentiles (25–75%) and range, respectively. The fold change of permeability compared with iBRB model itself before EBO-VLP administration is presented as the mean ±standard deviation. All values were determined in six independent experiments. Statistical analysis was performed using Student’s t test. **p < 0.01, ***p < 0.001.
(TIF)

S5 Fig Characterization of iBRB co-culture models by HRECs and HRPs. **Related to Fig 4.** (A) Assessment of integrity of co-culture iBRB in vitro barrier models with HRECs and HRPs by TEER every day in a week. The results are presented as the means ± standard deviation of six independent experiments. (B) Na-F permeability of iBRB co-culture models at 6 hours and 3 days post HREC seeding. The results are presented as the means ± standard deviation of four independent experiments. Statistical analysis was performed using Student’s t-test. (C) Images of HRECs showing expression of claudin-1, occludin, ZO-1 in co-culture with HRECs and HRPs. Claudin-1, occludin, ZO-1 are shown in green and cell nuclei stained with DAPI (blue). Representative images of three independent experiments are shown. The fluorescent Images were taken at 60× magnification objective lens under a confocal microscope. Statistical analysis was performed using Student’s t-test. ***p < 0.001.
(TIF)

S6 Fig Characterization of iBRB co-culture models by HRECs and HRAs. **Related to Fig 4.** (A) Assessment of integrity of co-culture iBRB in vitro barrier models with HRECs and HRAs by TEER every day in a week. The results are presented as the means ± standard deviation of six independent experiments. (B) Na-F permeability of iBRB co-culture models at 6 hours and 3 days post HREC seeding. The results are presented as the means ± standard deviation of four independent experiments. Statistical analysis was performed using Student’s t-test. (C) Images of HRECs showing expression of claudin-1, occludin, ZO-1 in co-culture with HRECs and HRAs. Claudin-1, occludin, ZO-1 are shown in green and cell nuclei stained with DAPI (blue). Representative images of three independent experiments are shown. The fluorescent Images were taken at 60× magnification objective lens under a confocal microscope. Statistical analysis was performed using Student’s t-test. ***p< 0.001.
(TIF)

S7 Fig Effect of EBO-VLP treatment on tri-culture VEGF-KD-iBRB models. **Related to Fig 4.** (A-B) siRNA-VEGF or nontargeting control siRNA were transfected into pericytes via Lipofectamine RNAiMax. (A) Total RNAs were analyzed by qRT-PCR. (B) Cell lysates were analyzed by western blotting using the anti-VEGF antibody and anti-β-actin antibody. Representative images of three independent experiments are shown.
(TIF)

S8 Fig Expression of claudin-1 in co-culture model and mono-culture iBRB models with EBO-VLP treatment. **Related to Fig 5.** (A) Western blot analysis of claudin-1 expression in HRECs at 48 h in the iBRB co-culture model with HRECs and HRPs. Representative images of three independent experiments are shown. (B) Western blot analysis of claudin-1 expression in HRECs at 48 h in the iBRB co-culture model with HRECs and HRAs. Representative images of three independent experiments are shown. (C) Western blot analysis of claudin-1 expression in HRECs at 48 h in the iBRB mono-culture model with HRECs. Representative images of three independent experiments are shown. (D) Relative claudin-1 levels of was normalized to β-actin. The results are presented as the means ± standard deviation of three independent experiments.
(TIF)

S9 Fig Distribution of VLP-ΔGP in retina of rat. **Related to Fig 7.** (A) H&E staining of retinas from rats without injection. Magnification: ×20. Representative images of three independent experiments are shown. (B) Immunohistofluorescence analysis of EBO-VLPs in the retinal tissue using anti-VP40 antibodies (red). The white arrow indicates EBO-VLPs. Representative images of three independent experiments are shown. The fluorescent images were taken with a 60× magnification objective lens under a confocal microscope.
(TIF)
