# Supplementary figures and images for: Ebola virus disrupts the inner blood-retinal barrier by induction of vascular endothelial growth factor in pericytes
Source: PLoS Pathog. 2023 Jan 18;19(1):e1011077. doi: 10.1371/journal.ppat.1011077 (PMC9847965; doi:10.1371/journal.ppat.1011077)

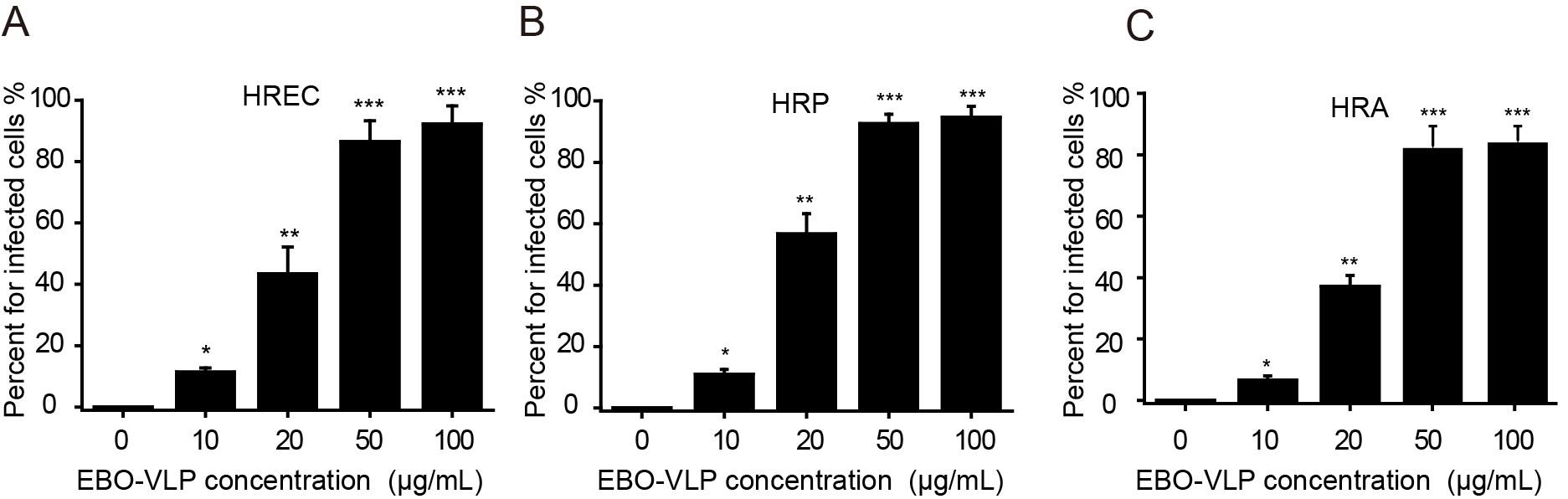

Supplement: S1 Fig — Related to Fig 1. (A-C) Percentage of infected cells including HRECs (A), HRPs (B) and HRA (C) with 0, 10, 20, 50 and 100μg/mL EBO-VLP treatment, respectively. The results are presented as the means ± standard deviation of three independent experiments. Statistical analysis was performed using Student’s t-test. All statistical analysis were compared with the 0μg/mL of EBO-VLP treatment, respectively. *p < 0.05, **p < 0.01, ***p < 0.001. (TIF) [file ppat.1011077.s002.tif]

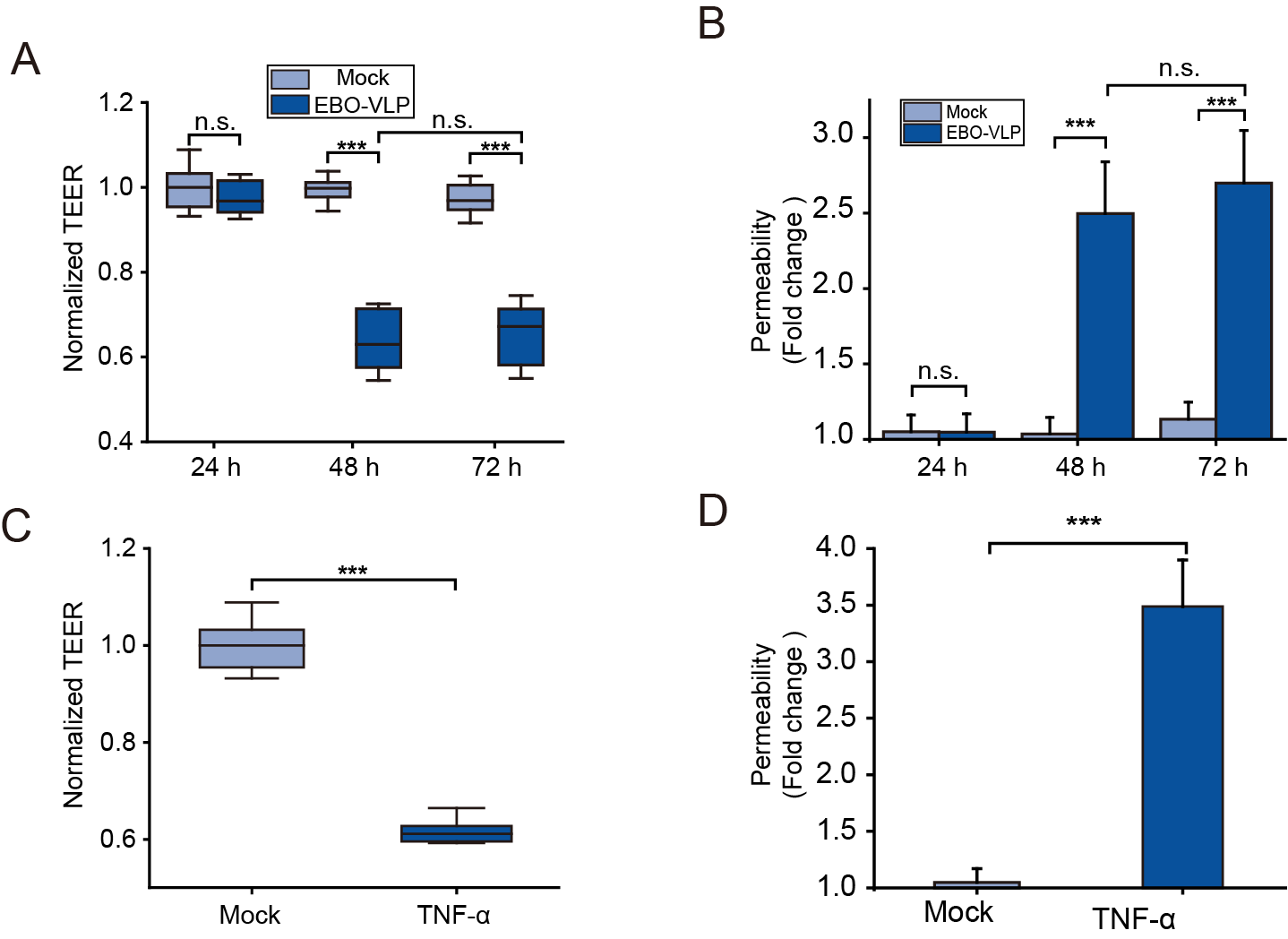

Supplement: S2 Fig — Related to Fig 1. (A-B) Integrity of the tri-culture iBRB model at 0 h, 24 h and 48 h post EBO-VLP administration. TEER values (A) and Na-F permeability (B) of the iBRB model were examined at 0 h, 24 h and 48 h after EBO-VLP administration. The box and the whisker present the median ± percentiles (25–75%) and range, respectively. The fold change of permeability compared with that of iBRB model itself before EBO-VLP administration is presented as the mean ± standard deviation. All values were determined in six independent experiments. (C-D) Integrity of the tri-culture iBRB model at 24 h post 10 ng/mL of TNF-α treatment. TEER values were normalized to those of iBRB models themselves before EBO-VLP administration. The fold change of permeability compared with that of iBRB model itself before EBO-VLP administration is presented as the mean ± standard deviation. All values were determined in six independent experiments. Statistical analysis was performed using Student’s t test. *p < 0.05, **p < 0.01, ***p < 0.001. (TIF) [file ppat.1011077.s003.tif]

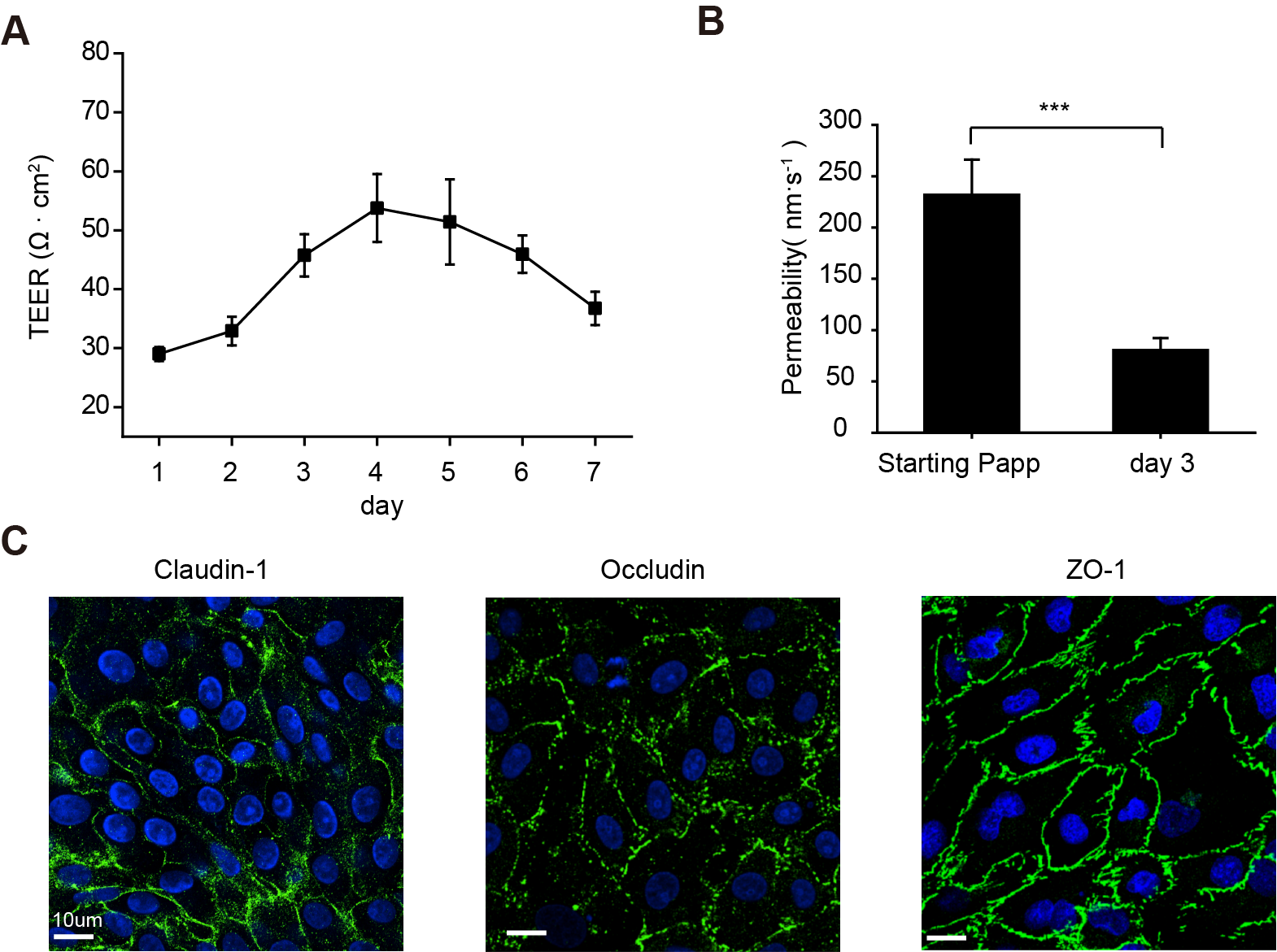

Supplement: S3 Fig — Related to Fig 2. (A) Assessment of integrity of mono-culture with HRECs in vitro barrier models by TEER every day in a week. The results are presented as the means ± standard deviation of six independent experiments. (B) Na-F permeability of iBRB mono-culture models at 6 hours and 3 days post HREC seeding. The results are presented as the means ± standard deviation of four independent experiments. Statistical analysis was performed using Student’s t-test. (C) Images of HRECs showing expression of claudin-1, occludin, ZO-1 in mono-culture with HRECs. Claudin-1, occludin, ZO-1 are shown in green and cell nuclei stained with DAPI (blue). Representative images of three independent experiments are shown. The fluorescent Images were taken at 60× magnification objective lens under a confocal microscope. Statistical analysis was performed using Student’s t-test. ***p < 0.001. (TIF) [file ppat.1011077.s004.tif]

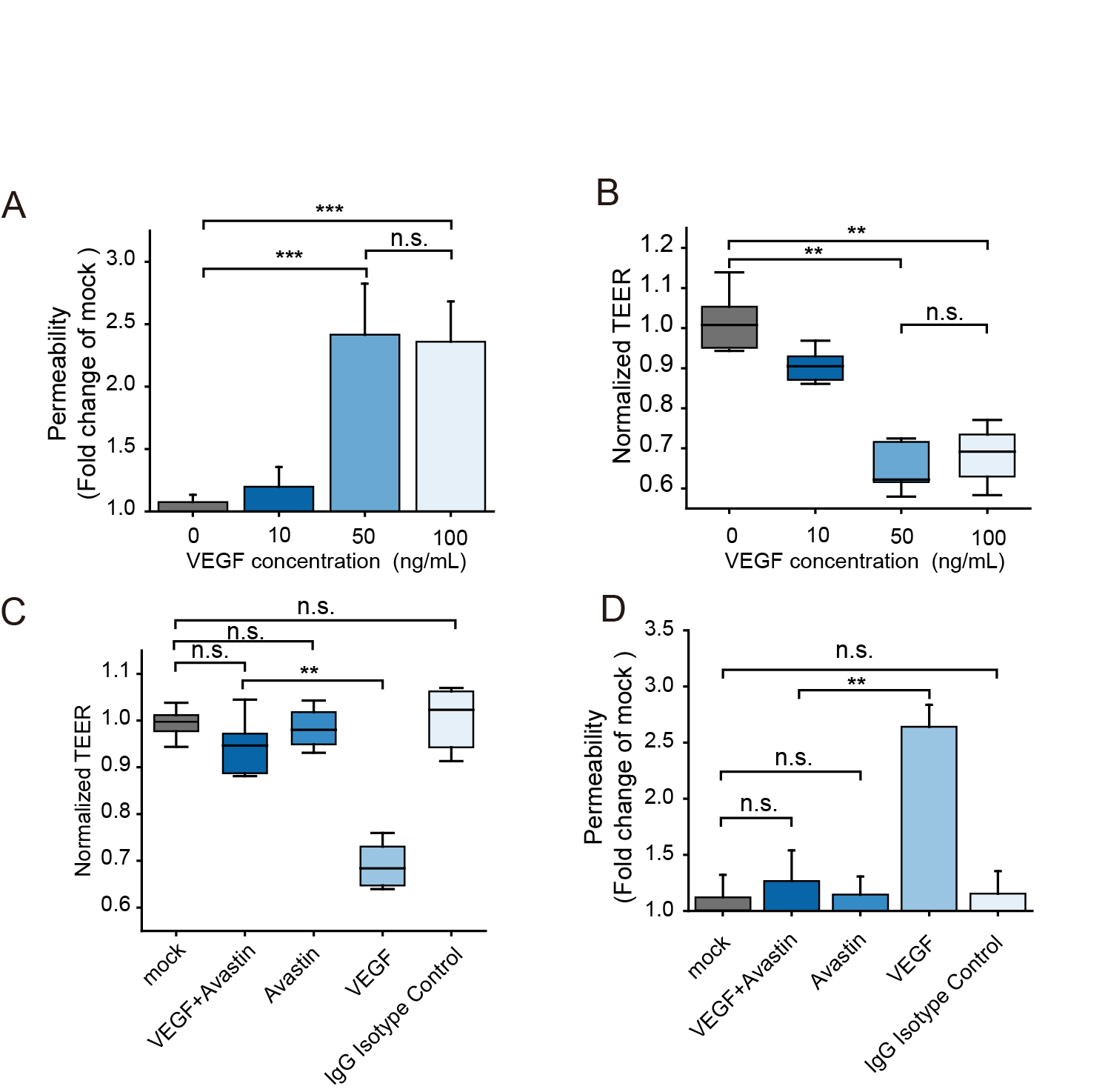

Supplement: S4 Fig — Related to Fig 4. (A-B) Integrity of the tri-culture iBRB model at different concentration of VEGF at 0,10, 50 and 100 ng/mL at 24 h. Na-F permeability (A) and TEER values (B) of the iBRB model were examined after VEGF administration. TEER values were normalized to those of iBRB models themselves before VEGF administration. The box and the whisker present the median ± percentiles (25–75%) and range, respectively. The fold change of permeability compared with that of iBRB model itself before EBO-VLP administration is presented as the mean ± standard deviation. All values were determined in six independent experiments. (C-D) Integrity of the tri-culture iBRB model treated with 50 ng/mL of VEGF, 100 ng/ml Avastin, 100 ng/mL of IgG isotype and 50 ng/mL VEGF + 100 ng/ml Avastin in 48 h. TEER values were normalized to those of iBRB models themselves before administration. The box and the whisker present the median ± percentiles (25–75%) and range, respectively. The fold change of permeability compared with iBRB model itself before EBO-VLP administration is presented as the mean ±standard deviation. All values were determined in six independent experiments. Statistical analysis was performed using Student’s t test. **p < 0.01, ***p < 0.001. (TIF) [file ppat.1011077.s005.tif]

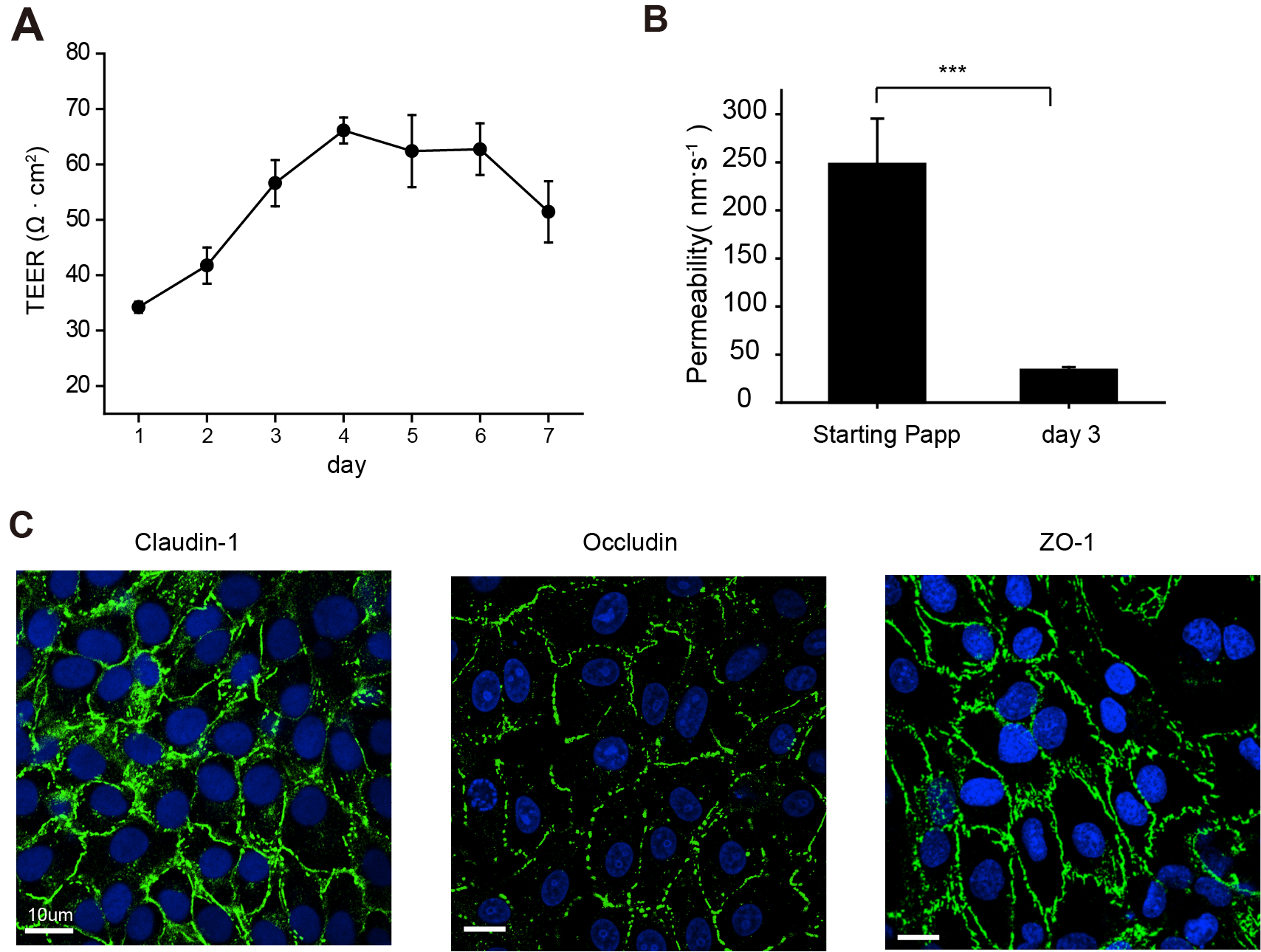

Supplement: S5 Fig — Related to Fig 4. (A) Assessment of integrity of co-culture iBRB in vitro barrier models with HRECs and HRPs by TEER every day in a week. The results are presented as the means ± standard deviation of six independent experiments. (B) Na-F permeability of iBRB co-culture models at 6 hours and 3 days post HREC seeding. The results are presented as the means ± standard deviation of four independent experiments. Statistical analysis was performed using Student’s t-test. (C) Images of HRECs showing expression of claudin-1, occludin, ZO-1 in co-culture with HRECs and HRPs. Claudin-1, occludin, ZO-1 are shown in green and cell nuclei stained with DAPI (blue). Representative images of three independent experiments are shown. The fluorescent Images were taken at 60× magnification objective lens under a confocal microscope. Statistical analysis was performed using Student’s t-test. ***p < 0.001. (TIF) [file ppat.1011077.s006.tif]

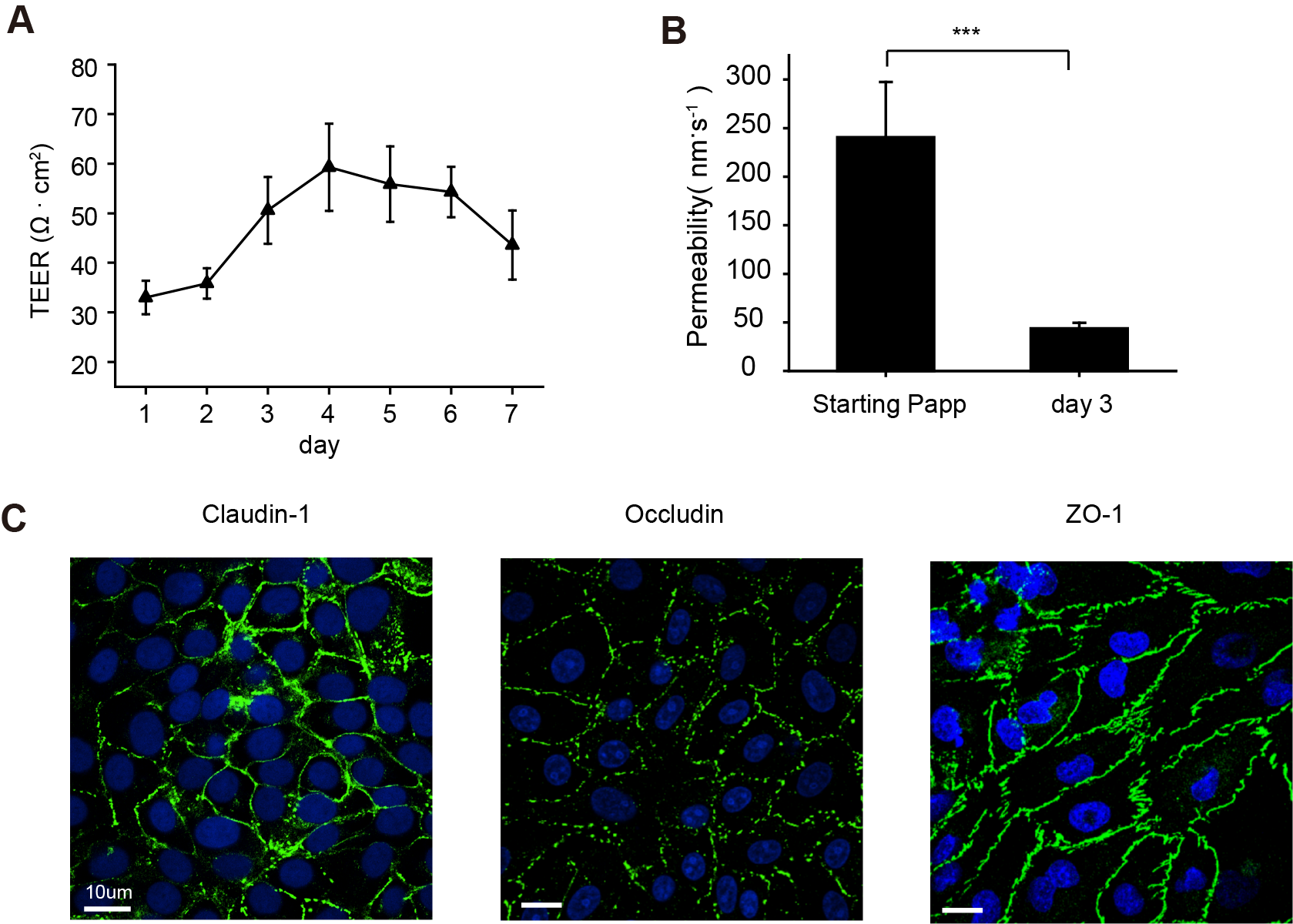

Supplement: S6 Fig — Related to Fig 4. (A) Assessment of integrity of co-culture iBRB in vitro barrier models with HRECs and HRAs by TEER every day in a week. The results are presented as the means ± standard deviation of six independent experiments. (B) Na-F permeability of iBRB co-culture models at 6 hours and 3 days post HREC seeding. The results are presented as the means ± standard deviation of four independent experiments. Statistical analysis was performed using Student’s t-test. (C) Images of HRECs showing expression of claudin-1, occludin, ZO-1 in co-culture with HRECs and HRAs. Claudin-1, occludin, ZO-1 are shown in green and cell nuclei stained with DAPI (blue). Representative images of three independent experiments are shown. The fluorescent Images were taken at 60× magnification objective lens under a confocal microscope. Statistical analysis was performed using Student’s t-test. ***p< 0.001. (TIF) [file ppat.1011077.s007.tif]

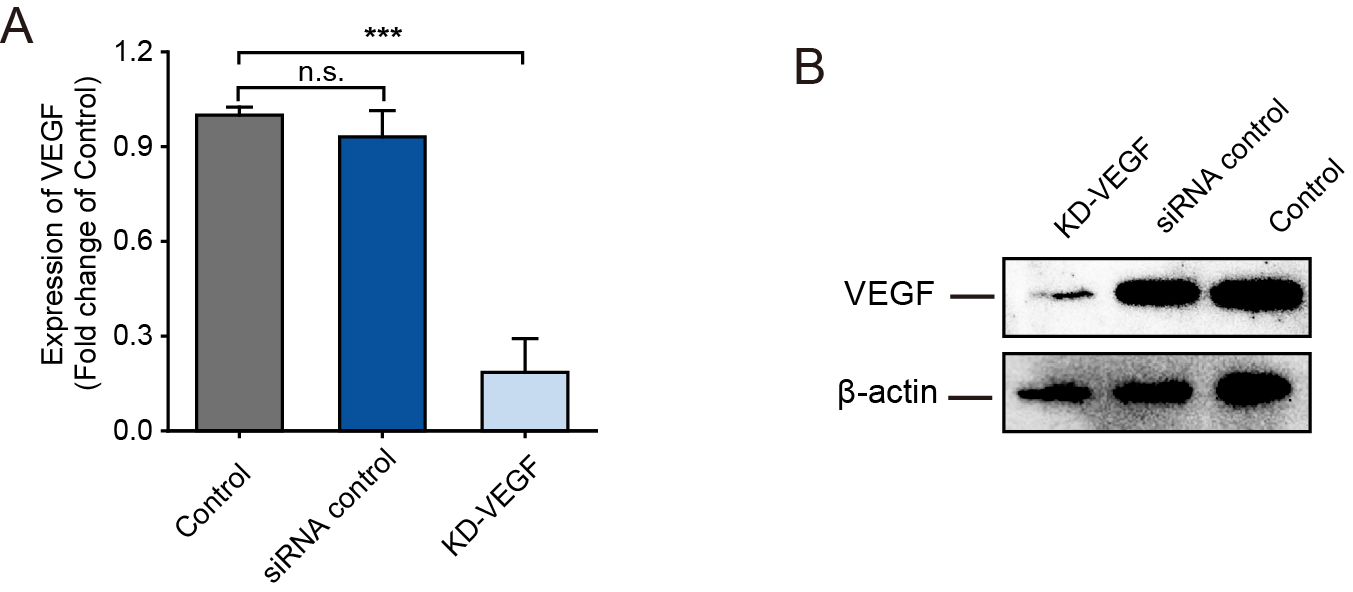

Supplement: S7 Fig — Related to Fig 4. (A-B) siRNA-VEGF or nontargeting control siRNA were transfected into pericytes via Lipofectamine RNAiMax. (A) Total RNAs were analyzed by qRT-PCR. (B) Cell lysates were analyzed by western blotting using the anti-VEGF antibody and anti-β-actin antibody. Representative images of three independent experiments are shown. (TIF) [file ppat.1011077.s008.tif]

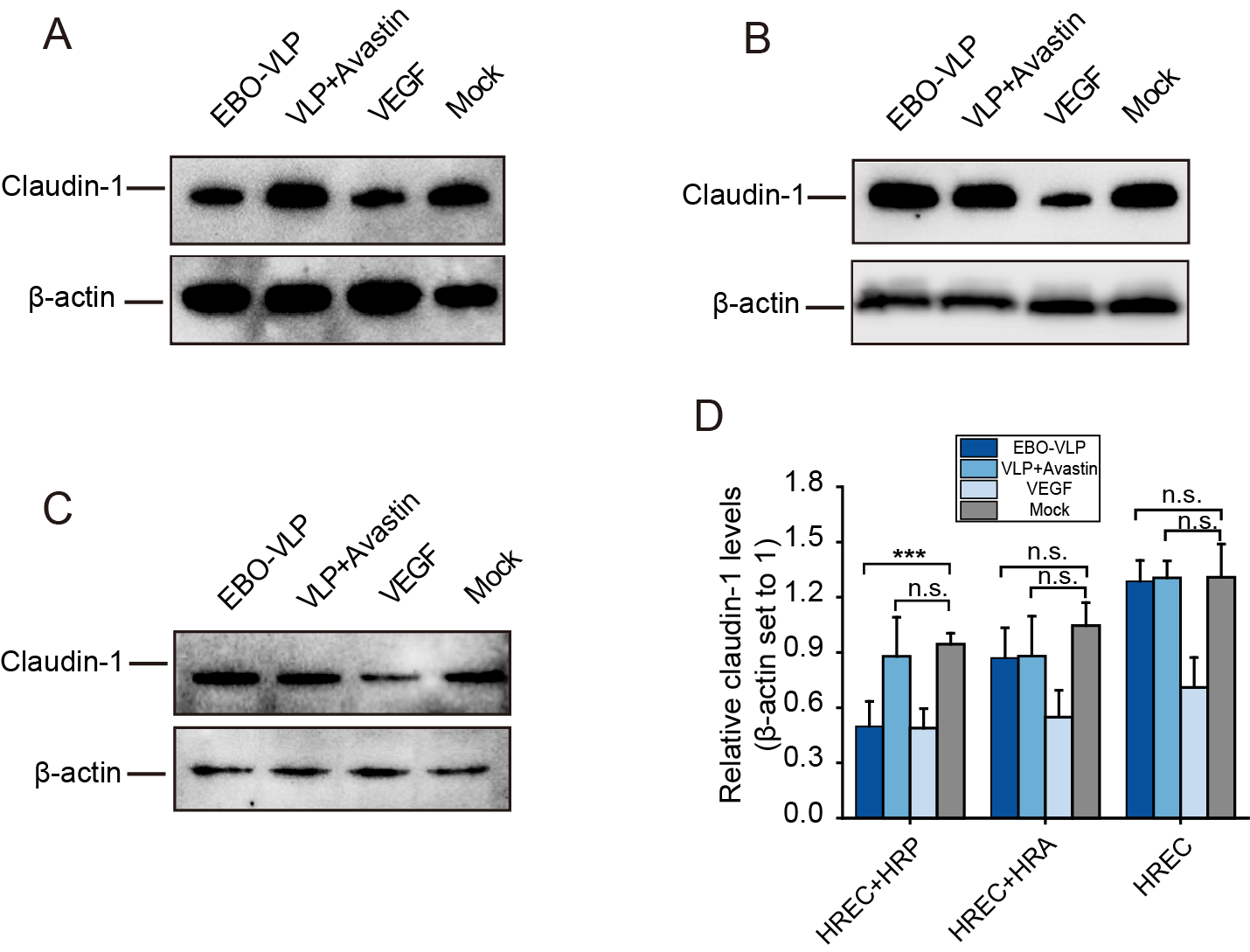

Supplement: S8 Fig — Related to Fig 5. (A) Western blot analysis of claudin-1 expression in HRECs at 48 h in the iBRB co-culture model with HRECs and HRPs. Representative images of three independent experiments are shown. (B) Western blot analysis of claudin-1 expression in HRECs at 48 h in the iBRB co-culture model with HRECs and HRAs. Representative images of three independent experiments are shown. (C) Western blot analysis of claudin-1 expression in HRECs at 48 h in the iBRB mono-culture model with HRECs. Representative images of three independent experiments are shown. (D) Relative claudin-1 levels of was normalized to β-actin. The results are presented as the means ± standard deviation of three independent experiments. (TIF) [file ppat.1011077.s009.tif]

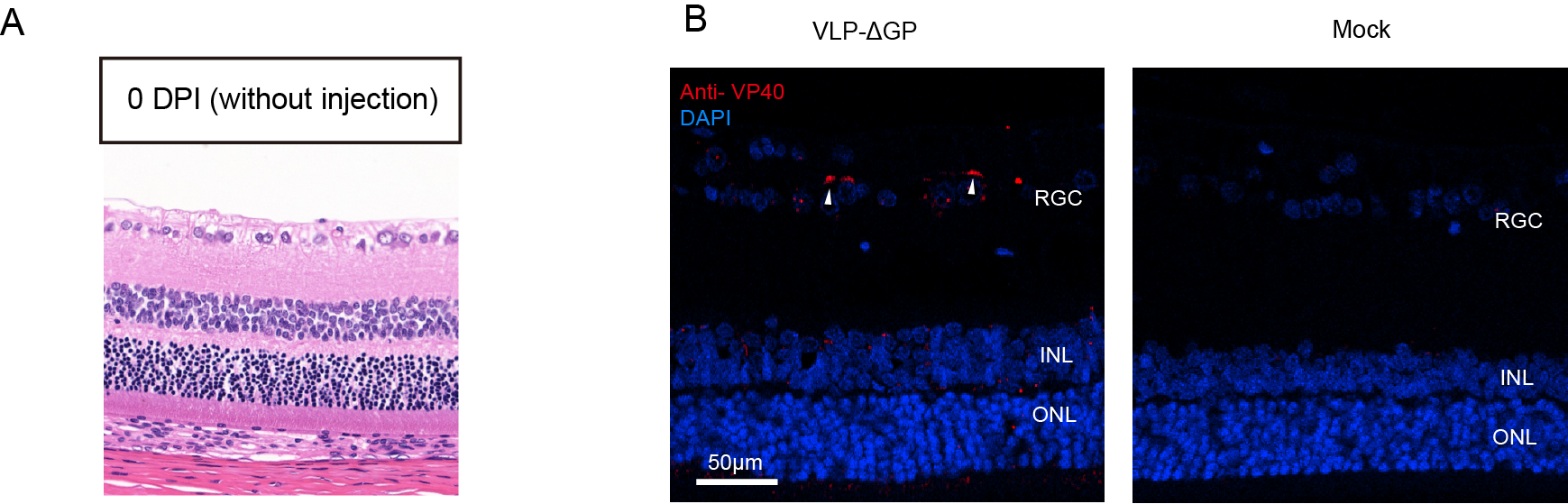

Supplement: S9 Fig — Related to Fig 7. (A) H&E staining of retinas from rats without injection. Magnification: ×20. Representative images of three independent experiments are shown. (B) Immunohistofluorescence analysis of EBO-VLPs in the retinal tissue using anti-VP40 antibodies (red). The white arrow indicates EBO-VLPs. Representative images of three independent experiments are shown. The fluorescent images were taken with a 60× magnification objective lens under a confocal microscope. (TIF) [file ppat.1011077.s010.tif]
